# A Set of miRNAs, Their Gene and Protein Targets and Stromal Genes Distinguish Early from Late Onset ER Positive Breast Cancer

**DOI:** 10.1371/journal.pone.0154325

**Published:** 2016-05-06

**Authors:** E. P. Bastos, H. Brentani, C. A. B. Pereira, A. Polpo, L. Lima, R. D. Puga, F. S. Pasini, C. A. B. T. Osorio, R. A. Roela, M. I. Achatz, A. P. Trapé, A. M. Gonzalez-Angulo, M. M. Brentani

**Affiliations:** 1 Oncology and Radiology Department, Laboratory of Medical Investigation 24 (LIM 24), University of Sao Paulo, Medical School, São Paulo, Brazil; 2 Laboratory of Medical Investigation 23 (LIM 23), Institute and Department of Psychiatry, University of Sao Paulo, Medical School, São Paulo, Brazil; 3 Mathematics and Statistic Institute, University of Sao Paulo, São Paulo, Brazil; 4 Department of Statistics, Federal University of Sao Carlos, São Paulo, Brazil; 5 Einstein Hospital, São Paulo, Brazil; 6 Department of Pathology of A.C. Camargo Cancer Center, São Paulo, Brazil; 7 Department of Oncogenetics of A.C. Camargo Cancer Center, São Paulo, Brazil; 8 Department of Breast Medical Oncology, The University of Texas M.D. Anderson Cancer Center, Houston, TX, United States of America; University of South Alabama Mitchell Cancer Institute, UNITED STATES

## Abstract

Breast cancer (BC) in young adult patients (YA) has a more aggressive biological behavior and is associated with a worse prognosis than BC arising in middle aged patients (MA). We proposed that differentially expressed miRNAs could regulate genes and proteins underlying aggressive phenotypes of breast tumors in YA patients when compared to those arising in MA patients. ***Objective***: Using integrated expression analyses of miRs, their mRNA and protein targets and stromal gene expression, we aimed to identify differentially expressed profiles between tumors from YA-BC and MA-BC. ***Methodology and Results***: Samples of ER+ invasive ductal breast carcinomas, divided into two groups: YA-BC (35 years or less) or MA-BC (50–65 years) were evaluated. Screening for BRCA1/2 status according to the BOADICEA program indicated low risk of patients being carriers of these mutations. Aggressive characteristics were more evident in YA-BC versus MA-BC. Performing qPCR, we identified eight miRs differentially expressed (miR-9, 18b, 33b, 106a, 106b, 210, 518a-3p and miR-372) between YA-BC and MA-BC tumors with high confidence statement, which were associated with aggressive clinicopathological characteristics. The expression profiles by microarray identified 602 predicted target genes associated to proliferation, cell cycle and development biological functions. Performing RPPA, 24 target proteins differed between both groups and 21 were interconnected within a network protein-protein interactions associated with proliferation, development and metabolism pathways over represented in YA-BC. Combination of eight mRNA targets or the combination of eight target proteins defined indicators able to classify individual samples into YA-BC or MA-BC groups. Fibroblast-enriched stroma expression profile analysis resulted in 308 stromal genes differentially expressed between YA-BC and MA-BC. ***Conclusion***: We defined a set of differentially expressed miRNAs, their mRNAs and protein targets and stromal genes that distinguish early onset from late onset ER positive breast cancers which may be involved with tumor aggressiveness of YA-BC.

## Introduction

The frequency of breast carcinoma (BC) diagnosed in young adults (YA) aged 35 years or less corresponds to 3–7% of the cases in western countries [**1]**, but may vary among different ethnic groups [**2–7**]. In Brazil the incidence of YA-BC accounts for 4% of total cases (Ministry of Health, SP, 2014).

Previous studies have suggested that YA-BC tumors exhibit clinical and pathological characteristics of a more aggressive disease, associated with a worse prognosis than BC in older patients [**1, 6, 8–10**]. According to Anders et al [**11**] the aggressive behavior of these tumors reflects the high frequency of triple-negative and HER-2 molecular subtypes in YA-BC. However, other studies proposed that breast cancer in young patients seems to be biologically distinct from that diagnosed in older patients, irrespective of BC distribution subtypes [**12, 13**].

Risk factors frequently reported as related to YA-BC include a familial history and presence of mutations in BRCA1/2, but these mutations could not explain most tumors arising in these patients. Recent work of our group showed that among YA-BC (N = 54) cases 31% were associated with familial history and 43% of these hereditary patients were carriers of BRCA 1/2 mutations, whereas these alterations accounted for only 9% of the sporadic cases [**14**]. Our results suggested that dysregulation in signaling pathways, other than those related to BRCA1/2 genes might be involved in the tumorigenesis of YA-BC.

Several studies have identified the involvement of putative signaling pathways entailed in the arising of YA-BC, irrespective of the BC subtype, such as growth factor-related pathways (mTOR, MAPK, PI3K/AKT, NF-kB, PTEN) as well as signaling cascades related to mammary stemness and immature cell populations (RANKL and c-kit) [**1, 8, 13, 15**]. **ColaK et al** [**16**] formulated a transcriptome analysis of breast tumors in young patients under 45 years and identified pathways related to cell cycle, embryonic development, DNA replication and proliferation.

An additional improvement of gene signatures could be expected from the determination of microRNAs that emerged as important players in BC [**17**]. MicroRNAs (miRs) are a group of small non-coding RNAs that regulates mRNA by binding to the 3’UTR region, inducing the mRNA degradation or repressing their translation into proteins. Thereby they control several biological processes related to proliferative homeostasis, differentiation and embryonic stemness [**18**]. Dysregulated miRs expression profiles perhaps point to diagnostic, prognostic, predictive and therapeutic applications [**19–22**]. Various studies described the roles of miRs as either tumor suppressors or oncogenes in breast cancers, depending on which genes or pathways they affect in a specific cellular context [**23**]. However, few studies addressed the role of miRs expression and their targets in BC occurring in early onset patients [**24, 25**].

Analysis of changes in mRNA levels induced by miRs suggested that repression of protein levels seem to reflect transcript levels [**26**]. Yet many targets are silenced by translational repression and measurements of miR effects at protein level are necessary to understand miR complex activities [**27**].

Integrative analyses of miRs expression profile and proteomics were used to assess changes in protein levels after the overexpression of selected miRs [**28**]. The authors concluded that miRs affected the expression of a great number of proteins. This approach was shown to be a useful tool to identify novel molecular mechanisms that mediate growth, development and progression of estrogen-dependent breast carcinomas [**29**]. Furthermore, functional proteomic analyses after artificial overexpression of specific miRs was effective to identify potential miR-regulated pathways that might be used for the development of new targeted therapies [**30**].

MicroRNAs have also been involved in the cross-talk between cancer cells and the tumor microenvironment [**31**]. Cancer-associated fibroblasts represent an important component of the tumor stroma [**32**] and are more abundant in advanced stages of breast cancer, correlated to poor prognosis [**33]**. Cancer associated fibroblasts have increased secretory ability, contributing to the release of growth factors, chemokines, cytokines and enzymes that promote angiogenesis, remodeling of extracellular matrix (ECM) and have immunosuppressive properties, possibly leading to enhancement of a more permissive microenvironment for tumor growth and progression [**34**]. A prior study indicated that the normal breast microenvironment of premenopausal women influences the behavior of breast cancer cells in vivo and in vitro [**35**]. Recently, it was suggested that stromal-related gene signatures may have prognostic significance in YA-BC, proposing that differences in microenvironment may account in part for age-specific differences in breast cancer behavior [**1**]. Nevertheless, little is known about differences in genes between the stromal components of YA-BC versus MA-BC tumors.

In this study, we proposed that differentially expressed microRNAs regulate genes and proteins underlying aggressive phenotypes of breast tumors in young patients as compared to those arising in middle age patients. A systems biology approach was suggested to explore the functional relationship among transcriptome, proteomic and metabolic alterations acquired by tumors and inputs from the environment that determine cellular behavior and biomarkers that will lead to the implementation of effective therapies [**36**]. Thus, we sought to perform an integrative analysis combining miRs, their target genes and proteins, as well as stromal genes expression, which provide complementary information aiming to identify profiles differentially expressed between breast carcinomas of young adult patients (YA-BC) compared to breast carcinoma samples of middle age patients (MA-BC). We focused our comparison on hormone receptor positive tumors. To address the contribution of the stroma (primarily fibroblasts) to the biological differences between breast carcinomas from both groups we used laser capture microdissection (LCM) to isolate tumor stroma. A microarray analysis then identified differences in the stromal gene expression profiles of YA-BC and MA-BC tumors.

## Material and Methods

### Patients

Fifty frozen tumor tissues provided by A. C. Camargo Biobank (Sao Paulo, SP, Brazil) were collected from 2011 to 2014. They were evaluated, 25 from breast cancer patients aged 35 years or less and 25 from patients 50–65 years old undergoing surgery. Samples were retrospectively collected after signing an informed consent form authorizing the use in research. This study was approved by the Ethical Board for Research Project Analysis (CEP) of A. C. Camargo Cancer Center (1656/12) and was conducted in accordance to the Helsinki Declaration. The inclusion criteria were: 1) young adult women aged ≤ 35 years (YA-BC) and middle aged women between 50–65 years (MA-BC) with invasive ductal breast carcinoma; 2) sporadic cases with low risk of germline mutation of BRAC1/2 genes and 3) tumors displaying positive hormone receptors. Sporadic cases characterize patients that do not meet the criteria of NCCN (National Comprehensive Cancer Network) and do not present a history of first-degree relatives or more than two members of the 2nd degree diagnosed before 35 years of age. Risk of BRCA1/2 genes mutations were calculated by the *Breast and Ovarian Analysis of Disease Incidence and Carrier Estimation Algorithm* (BOADICEA) database, based on the family pedigree data, which include: number of affected individuals in the family as well as patient ages, deaths, cancer types and ages at diagnosis of each family individual. Patients undergoing neoadjuvant treatment were excluded.

Clinical and pathological descriptors of these patients, including pathological features of the tumor specimens were collected: age, pregnancy frequency, use of contraceptive pills, histological type, disease staging (TNM) at diagnosis, and histological grade. *Scarff-Bloom-Richardson* (SBR) modified from Nottingham Histologic Scoring method was used to classify histological grade. Tumor size was calculated microscopically by histological slides from the representative microscopic cutting through the surgical piece. Tumors < 2 cm were classified as T1 and > 2 cm tumors were classified as T2, T3. Estrogen receptor (ER), progesterone receptor (PR), and Her-2 status were determined by immunohistochemistry. Only tumor samples with distinct nuclear immunostaining in ≥10% of the cells were recorded as ER-positive. For PR positivity results were registered when 20% of the nuclei were moderately to strong stained [**37**]. The positive status for Ki67 was granted to cases with ≥ 14% moderate to strongly stained nuclei [**38**]. Her-2 status was considered positive if the membrane staining reaction was defined as 3+. In doubtful cases (2+), fluorescence *in situ* hybridization (FISH) was additionally performed. Tumors were further classified as luminal A or B. In this study, the classification of BC subtypes was determined by immunohistochemistry, according to the Surrogate definitions of molecular subtypes of breast cancer defined during the 13^th^ ST Saint Gallen International Breast Cancer Conference, 2013. The following definitions were used to determine the tumor surrogate luminal subtypes: the luminal A subtype exhibiting ER and PR positive, Her-2 negative and low Ki67 (< 14%); the luminal B exhibiting ER positive, HER-2 negative and high Ki67 (≥14%) and/or PR negative (< 20%); luminal B can also exhibit ER positive, HER2 positive and any ki67 and any PR [**37**].

### Total RNA and microRNA isolation

Frozen tumor tissues (approximately 30 mg) were homogenized with the Precellys 24^®^ equipment (Carlsbad, California, USA). The supernatant was used to purify total RNA and microRNA with the miRNeasy Mini kit (#217004, Qiagen, Venlo, the Netherlands) and the isolation was automatically performed by QIAcube® (Qiagen) according to the manufacturer's protocol. Total RNA and microRNA qualities and concentrations were assessed using ND-1000 NanoDrop™ (Thermo Scientific, Wilmington, Delaware, USA) and the integrity was determined using an Agilent Bioanalyzer 2100 (Agilent Technologies, Palo Alto, California, USA).

### MicroRNA expression profiling

A global profiling of miR expression in these 50 tumor samples was performed using the TaqMan Low Density Array Human microRNA assay panel A (TLDA, Applied Biosystems). The array panel A contains 377 *homo sapiens* miRs and seven endogenous controls (ribosomal RNAs) for a total of 384 probes. Reverse transcription was performed with the RT-miRs kit and the pre-amplification with the pre-amplification kit (Applied Biosystems) using 100 ng of miRNA according to the manufacturer’s protocols. Real time PCR (RT-PCR) was performed according to MIQE guidelines [**39**] following the 7900 HT Real Time PCR Systems protocol using 2× Universal PCR Master Mix, no AmpErase UNG.

The expression value, measured as cycle threshold (CT), of each miR was obtained using SDS 1.2 software (Applied Biosystems: TaqMan^®^ OpenArray^®^ Real-Time PCR Plates). MiRs presenting expression levels below the detection limit (>38) in more than 60% of samples were excluded from analyses. To calculate the expression of miRs for each tumor sample, the delta CT method was used and normalization was performed with the median of RNU44, RNU48 and MammU6 endogenous controls assays (CT of miR—CT of endogenous). The miRs expression levels were calculated by 2^−ΔCT^ for tumor samples from 25 tumors of the YA-BC group and 25 tumors of the MA-BC group.

### Target prediction

Putative targets were inferred for each miR using the miRWalk prediction program database algorithm to extract predictions from TargetScan, RNA22, DIANAmt, miRanda, miRBD, PITA and PicTar (http://www.umm.uni-heidelberg.de/apps/zmf/mirwalk/index.html). The final miR target prediction results were a combination of the queries and the predictions occurring in at least 3 of these 7 databases. Targeting criteria were as follows (a) near-perfect complementarity in the 7–8 nt region close to 5′-end of the miR (seed sequence) with the 3′-UTR region of the target sequence; (b) conserved target sequence sites between species; (c) strong thermodynamic stability of miR:mRNA duplex; (d) complementarity between multiple sites; (e) existence of a central non-matched region (loop).

The final selection of target candidates was established by combining genes predicted by the miRWalk database and also exhibiting differential expression with ≥ 80% of confidence level from the microarray experiment profile. Thus, inverse or concordant regulation of these targets by at least one of our eight miRs was considered. The nomination of miR:mRNA inverse regulation was considered when miRs play a negative regulatory role on their mRNA targets; given that the miR:mRNA pairs meeting the condition that the expression levels of the genes exhibited inverse regulation with their corresponding miRs. In other words, if a given miR was up regulated, the expression of its target is expected to be down regulated and vice-versa. The nomination of miR:mRNA concordant regulation was considered when miRs play a positive regulatory role on their mRNA targets; given that the miR and its target gene were altered in the same direction [**40**].

### Gene expression profiling

A microarray assay was performed based on two-colors labeled cDNAs hybridized to the Agilent GE 8X60K G4851A whole human genome oligoarray (Agilent, Santa Clara, USA). Expression data (log2) from each sample, and signals of hybridization controls, were extracted, as background and low expression signals of more than 30% of samples in the corresponding group were removed from the analyses. The mean of the probe replicates were calculated, and next, the normalization between samples was performed by *limma library R version 2*.*13*. All microarray raw data have been deposited in the GEO public database (http://www.ncbi.nlm.nih.gov/geo/GSE77358), a MIAME compliant database.

### Protein expression profiling

Protein extraction was performed using protocols from Functional Proteomics RPPA Core Facility at MD Anderson Cancer Center. Frozen tissue samples were cut into small pieces, stored on dry ice, and then homogenized with precellys (#9806, Cell Signaling Technology, Danvers, MA, USA) that contained fresh protease/phosphatase inhibitors (#5872, Cell Signaling Technology). The lysate was centrifuged for 10 min at 14,000g in a cold microfuge, and the supernatant was collected for RPPA analysis. Protein concentrations were determined using BSA reagents (Thermo Scientific/Pierce, Rockford, IL, USA). Aliquots of total tissue frozen lysates were sent to MD Anderson Cancer Center (Houston, TX, USA). The protein expression determination was performed by Reverse Phase Protein Array (RPPA) at the Functional Proteomics RPPA Core Facility at MD Anderson Cancer Center, as previously described [**41**]. Each slide was probed with a validated primary antibody adding a biotin-conjugated secondary antibody. After scanning, spot intensity of the stained slides was obtained from each spot. All data were normalized for protein-loading correction factor and transformed into linear values [**42, 43**].

This RPPA analysis included a panel of 130 antibodies detecting total protein and/or its activated forms (such as phosphorylated or cleaved proteins). The antibody panel reacted with key proteins involved in critical signaling pathways and networks, such as the phosphatidylinositol 3-kinase/AKT pathway, the extracellular signal-regulated kinase (ERK)/ mitogen-activated protein kinase pathway, the Janus kinase (JAK)/signal transducers and activators of the transcription (STAT) pathway, the tyrosine kinase pathway, cell death and survival, cell-cycle regulation, cell growth and proliferation, and cellular movement.

### Functional and pathways enrichment analyses

Over-representation of pathways and biological process in the differentially expressed targets and proteins was determined with ***WEB-based GEne SeT AnaLysis Toolkit* (**WebGestalt) based on KEGG and Gene Ontology (GO) annotations. Statistical tests from Webgestalt webpage include the χ2 test, the T test, the binomial test and the hypergeometric test, which gives greater set of biomarkers possibilities, rather than miss some false negative genes.

### Interatoma analyses

The analysis was performed using the protein-protein interatoma String program that integrates other interaction databases [44]. In this analysis we consider protein-protein interactions of various types, including genetic and physical (mapping corresponding protein products), as determined by a variety of methods. This index allows the user to search for a protein and recover a non-redundant list for this interacting protein whose interaction was considered as first level, or a protein that directly interacts with another protein without need for other protein complexes. The String program was used to construct and visualize molecular interaction networks.

### Statistical analysis

Comparisons statistics of miRs expression between YA-BC and MA-BC were conducted with focus on the medians of each population of expression. This technique is described by Pereira et al [**45**]. It corresponds to finding two order statistics, one for YA-BC and another for MA-BC: These order statistics should differ and the highest order among the two should produce a smaller statistical value than the value of the order statistics of the other group. It is calculated so that the probability of the population median of the first group is smaller than the order statistics (larger order) chosen for this sample multiplied by the probability that the population median of the second group is greater than the statistical order (smaller order) of that second group. Consequently, we conjecture that the median of the first population is lesser than the second. Next we assessed the probability that the conjecture is true. Of course, the choice of these statistics depends on the sample result and the calculation of probabilities is over the population events. As in the case of confidence intervals, one changes the word probability for confidence. If an event is very likely to occur and we do not know that it actually occurred in the sample we can say, for example, that if it is very likely that an event occurs, why did it not occur in our sample? That is the real reason for the change of probability for confidence. In short, after observation of the samples, we consider the confidence in the claim that the median of the first population is smaller than that of the second. With a cutting indicator defined when miRs are differentially expressed between YA-BC and MA-BC, it also helps to decide which of the two populations has significantly higher (or lower) value of the medians. The procedure for selecting differentially expressed genes and proteins was the same. Here we describe the technique using molecules to represent both. After finding differentially expressed molecules, pairs of under and over expressed molecules for setting an index (indicator able to classify the individual samples in appropriate groups) were selected to simulate ratings. The best pairs were chosen among those with high confidence and previously linked to cancer. To find out how many pairs are needed to comprise the index, we sequentially used one pair, two pairs, and so on. The stopping rule was when the set of pairs gives us a high accuracy in classification and meets the classification quality objectives. This index is calculated as the product of the ratios between the highest and lowest values ​​of each selected pair. The accuracy of classification is obtained by the sensitivity and specificity values ​​using the leave-one-out technique [**46**]

Statistical analysis of the clinicopathological data to describe the frequency and discriminate the significant differences between tumors YA-BC and MA-BC group was based on chi-square test. To define over and under-expression in this analysis we used the ROC curve (receiver-operating characteristic) and the AUC (area under the curve). All analyses that resulted in a p-value ≤ 0.05 were considered statistically significant.

### Microenvironment gene expression profile analysis

Laser Capture Microdissection (LCM) was performed to isolate enriched stromal (ES) cells, (primarily fibroblasts within the stroma). The isolation kit (PicoPure RNA) from Arcturus was used to isolate total RNA according to the manufacturer's protocol. After extraction, the concentration of total RNA was determined by reading the absorbance at 260 and 280nm by spectrophotometer NanoDrop ND-1000 UV-Vis Spectrophotometer (NanoDrop Technologies, Agilent), the purity was determined by the ratio between values of 260/280. Around 100pg of total RNA isolated from ES was submitted to RNA amplification (*Arcturus kit* # 02102012), followed by microarray assay (Agilent GE 8X60K G4851A whole human genome oligoarray; Agilent, Santa Clara, USA).

## Results

### Patients

Samples were categorized into two groups: YA-BC and MA-BC. The median age of patients was 31 years (minimum 23, maximum 35) for the YA-BC group and 57 years (minimum 50, maximum 64) for the MA-BC group.

### Clinicopathological features of tumors

Among the twenty-five tumors of YA-BC group we observed an incidence of 32% of luminal A and 68% of luminal B. The expression of cytokines 5,6,14 was negative for all cases. Moreover, 36% of YA-BC tumors were classified as stage III or IV, and 48% to histological grade 3 (**Table 1**). Only 10 patients had information about the mitotic index, which was higher than 10 for 80% of YA-BC patients. When clinicopathological features were compared in the YA-BC and MA-BC groups, no significant differences were observed between the luminal subtypes A and B (p = 0.769) and the age at diagnosis, while no significant associations were manifested in relation to the mitotic index and compromised lymph nodes. A significant correlation between YA-BC and other indicators of aggressive phenotype was observed, and defined by larger tumor size (p < 0.001) and higher histological grade (p = 0.047) that occurred in greater proportion in the YA-BC group. No significant associations were observed between use of contraceptive pills, pregnancy, and smoking in YA-BC and MA-BC groups. Risk for BRCA1/2 mutations was low (<15%) for 25 patients from YA-BC group evaluated by the BOADICEA to be mutation carriers. Sequence analyses of 5 tumors of patients that have been sequenced in a previous work of our group [**25**] confirmed the absence of mutation risk corroborating BOADICEA analysis.

**Table 1:** Clinicopathological characteristics of tumors in YA-BC versus MA-BC groups.

Data	YA-BC (≤35years)	MA-BC (50-65years)	p-value
	n(%)	n(%)	
**Molecular classification**			
Luminal A	8 (32)	10 (40)	0.769
Luminal B	17 (68)	15 (60)	
**TNM**			
I and II	16 (64)	22 (88)	0.095
III and IV	9 (36)	3 (12)	
**Lymphnodes**			
Positive	12 (75)	19 (79)	0.525
Negative	4 (25)	5 (21)	
**Histological grade**			
1 and 2	13 (52)	15 (79)	**0.047**
3	12 (48)	4 (21)	
**Mitotic index**			
<10 CGA	2 (20)	2 (15)	0.596
≥10 CGA	8 (80)	11 (85)	
**Tumor size**			
<2 cm	9 (39)	22 (88)	**<0.001**
≥2 cm	14 (61)	3 (12)	
**Contraceptive pill use**			
No, never	4 (36)	8 (38)	0.617
Yes, for at least 1 year	7 (64)	13 (62)	
**Cigarette smokers**			
No, never	12 (86)	11 (61)	0.127
Yes	2 (14)	7 (39)	
**Pregnancy**			
None	8 (47)	8 (36)	0.364
At least once	9 (53)	14 (64)	

### MicroRNA expression profile

Quantitative real-time PCR showed that 68 out of 377 miRs had CT values ≥ 39 in more than 60% of the samples and, therefore, were excluded from further analyses. Eight microRNAs were differentially expressed between YA-BC and MA-BC (high confidence statement larger than or equal to 90%) (**Table 2**) and most of these miRs were identified as up-regulated (miR-9, miR-210, miR-106a, miR-106b, miR-18b, miR-33b and miR-518a-3p) whereas only one miR (miR-372) was found to be down-regulated in YA-BC compared to MA-BC. The expression levels of miR-9, miR-33b, miR-210, miR-106b, miR-518a-3p and miR-372 were correlated with a more aggressive phenotype as indicated by tumor size and TNM stage (p-value < 0.05) (**Table 3**). The over-expression of miR-9, miR-33b, miR-210 and under-expression of miR-372 was significantly associated with large tumor size (≥ 2cm) (p = 0.038; p = 0.016; p = 0.015; p < 0.001, respectively). The under-expression of miR-106b and miR-518a-3p was significantly associated with a less advanced stage (TNM I and II) (p <0.001; p = 0.02 and p = 0.002, respectively (**Table 3**).

**Table 2:** Differentially expressed miRs (YA-BC *vs* MA-BC).

MiR	Statement	Confidence	Sequence of miR#	Genomic region#	Genomic position#
** hsa-miR-9**	YA > MA	0.99	UCUUUGGUUAUCUAGCUGUAUGA	intergenic	1q22
** hsa-miR-106a**	YA > MA	0.97	AAAAGUGCUUACAGUGCAGGUAG	intergenic	Xq26
** hsa-miR-106b**	YA > MA	0.94	UAAAGUGCUGACAGUGCAGAU	intragenic	7q22
**hsa-miR-18b**	YA > MA	0.94	UAAGGUGCAUCUAGUGCAGUUA	intergenic	Xq26
** hsa-miR-210**	YA > MA	0.93	CUGUGCGUGUGACAGCGGCUGA	intergenic	11p15
** hsa-miR-33b**	YA > MA	0.93	GUGCAUUGCUGUUGCAUUGCA	intragenic	17p11
** hsa-miR-518a-3p**	YA > MA	0.93	GAAAGCGCUUCCCUUUGCUGGA	intergenic	19q13
**hsa-miR-372**	YA < MA	0.93	AAAGUGCUGCGACAUUUGAGCGU	intergenic	19q13

# Information according to the miRBase. Over expression of miR in YA-BC group (YA > MA); under expression of miR in YA-BC group (YA < MA).

**Table 3:** Association between miRs and tumors clinicopathological characteristics.

miR	Tumor size			TNM		
	< 2 cm	≥ 2 cm	p-value	I and II	III and IV	p-value
**miR-9**						
under	21 (78%)	6 (22%)	0.038	26 (96%)	1 (4%)	< 0.001
over	10 (48%)	11 (52%)		12 (52%)	11 (48%)	
**miR-18b**						
under	18 (78%)	5 (22%)	0.075	19 (79%)	5 (21%)	0.75
over	13 (52%)	12 (48%)		19 (73%)	7 (27%)	
**miR-33b**						
under	21 (81%)	5 (19%)	0.016	23 (85%)	4 (15%)	0.18
over	10 (46%)	12 (55%)		15 (65%)	8 (35%)	
**miR-106a**						
under	19 (76%)	6 (24%)	0.13	20 (80%)	5 (20%)	0.74
over	12 (52%)	11 (48%)		18 (72%)	7 (28%)	
**miR-106b**						
under	16 (73%)	6 (27%)	0.37	21 (91%)	2 (9%)	0.02
over	15 (58%)	11 (42%)		17 (63%)	10 (37%)	
**miR-210**						
under	20 (83%)	4 (17%)	0.015	19 (79%)	5 (21%)	0.75
over	11 (46%)	13 (54%)		19 (73%)	7 (27%)	
**miR-372**						
under	8 (38%)	13 (62%)	<0.001	15 (65%)	8 (35%)	0.18
over	23 (85%)	4 (15%)		23 (85%)	4 (15%)	
**miR-518a-3p **						
under	15 (65%)	8 (35%)	1.0	23 (96%)	1 (4%)	0.002
over	16 (64%)	9 (36%)		15 (58%)	11 (42%)	

Under expression of miRs (under); over expression of miRs (over) in the YA-BC as compared to MA-BC group.

### Integrated analysis of miRNA and mRNA expression profiles

*In silico* analysis resulted in a total of 38.974 mRNAs predicted to be the targets for the eight miRNAs differentially regulated between YA-BC and MA-BC. For each miRNA, 972 to 11.711 targets were identified, while miR-9 presented the highest number of predicted mRNA targets. Furthermore, most of these targets were found to be commonly regulated by more than one of the eight miRNAs.

#### mRNA-target expression profile by microarray

Microarray analyses showed 1306 mRNAs differentially expressed, with 517 being up-regulated and 789 down-regulated in YA-BC compared to MA-BC. Integrated analysis of these genes with the 38974 target mRNAs predicted by *in silico* brought about 602 genes potentially regulated by at least one of the eight miRNAs differentially expressed between YA-BC and MA-BC (**S1 Table**). We have identified 440 inverse miRNA:mRNA regulatory relationships i.e. increased miRNA expression corresponded to under-expression of the respective mRNA, and vice-versa. Among these interactions, 363 genes showed decreased expression, whose regulatory miRNA were over-expressed while 77 genes were identified as up-regulated and the correspondent miRNAs were under-expressed in YA-BC when compared to MA-BC. The remaining 162 differentially expressed genes predicted to be microRNA targets showed concordant relationship with their regulatory miRNA, suggesting a non-canonical regulatory mechanism. Webgestalt analyses of the 602 target genes revealed involvement in several over represented pathways including metabolism (P = 0.002), MAPK signaling (P = <0.001), neurotrophyns (p = 0.002) and Oocyte meiosis signaling (P<0.0006).

**Fig 1:**
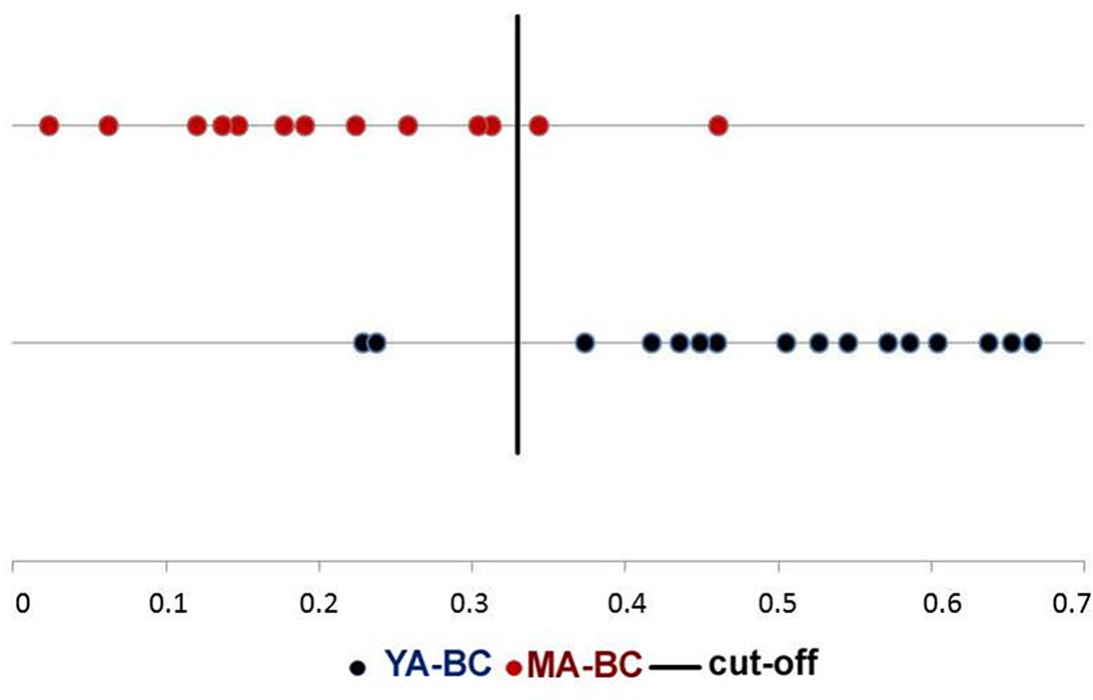
Sample classifications based on genes. Four index pairs of genes as potential classifiers of samples (circles) into the right side of cut-off mark corresponding to YA-BC group, and to the left side corresponding to MA-BC.

Four index pairs composed by eight mRNAs inversely regulated by the eight miRNAs (***ESR1*, *RPS6KA1*, *YWHAZ*, *BCL7*, *PARP12*, *DUSP2*, *DUSP8* and *PIGS***) were chosen for samples classification. The individual samples were correctly classified with 88% and 85% of confidence, respectively to be placed in the YA-BC or MA-BC group (**Fig 1).** Correlation analyses between these genes and clinicopathological features indicated that under-expression of PARP12 in YA-BC showed a significant association with positive lymph nodes (p = 0.03) (**S2 Table**).

### MiRNA-regulation protein-protein interaction (PPI)

Total proteins were extracted from some samples included in previous analyses: 21 tumor samples from the YA-BC group of patients and nine from the MA-BC group. The RPPA data set consists of 210 proteins, and after analysis of expression levels, 24 proteins were differentially expressed comparing tumors of both groups. These 24 proteins might be regulated by at least one of the eight miRs ensuring from this study, once their corresponding genes were predicted as target genes by the *in silico* analysis, and therefore were named miR-protein (**Table 4**). Of the 24 proteins mentioned above, 15 miR-proteins pairs followed inverse regulation, that is to say, if the miR showed a reduction, the corresponding protein showed an increase in expression or vice versa. The remaining nine miR-protein pairs, although differentially expressed, did not follow this inverse regulation suggesting a coordinate regulation.

**Table 4:** 24 proteins regulated by at least one of the eight miRs.

Protein	Protein name	Statement_protein	Confidence_protein	regulator miR
EIF4E	*eukaryotic translation initiation factor 4E *	**YA < MA**	0.9783	**↑ miR-9**
RAD50	** ***RAD50 homolog S*.*cerevisiae*	**YA < MA**	0.9493	**↑ miR-9; 33b**
STAT5A	*signal transducer and activator of transcription 5A *	**YA < MA**	0.9081	**↑ miR-18b; 33b**
ESR1	*estrogen receptor*	**YA < MA**	0.8986	**↑ hsa-miR-9; 18b; 33b; 106a/b; ↓372**
PXN	*Paxillin*	**YA < MA**	0.8986	**↑ hsa-miR-9**
EIF4EBP1	*eukaryotic translation initiation factor 4E binding protein 1 *	**YA < MA**	0.8812	**↑ miR-18b**
CTNNB1	*catenin (cadherin-associated protein)*, *beta 1*, *88kDa *	**YA < MA**	0.8812	**↑ miR-33b**
GYS1	*glycogen synthase 1 (muscle) *	**YA < MA**	0.8812	**↑ miR-106a/b; ↓372**
MDM2	*Mdm2*, *p53 E3 ubiquitin protein ligase homolog (mouse) *	**YA < MA**	0.8812	**↑ miR-106a/b; ↓372**
RPS6KA1	*ribosomal protein S6 kinase*, *90kDa*, *polypeptide 1 *	**YA < MA**	0.8812	**↑ miR-106a/b; ↓372**
RPS6KA2	*ribosomal protein S6 kinase*, *90kDa*, *polypeptide 2 *	**YA < MA**	0.8812	**↑ hsa-miR-9; 33b; 106a/b; 518a-3p; ↓372**
RPS6KA3	** ***ribosomal protein S6 kinase*, *90kDa*, *polypeptide 3*	**YA < MA**	0.8812	**↑ hsa-miR-9; 18b; 33b; 106a/b; 518a-3p; ↓372**
NDRG1	*N-myc downstream regulated 1 *	**YA < MA**	0.8341	**↑ hsa-miR-9; 18b**
RAF1	*v-raf-1 murine leukemia viral oncogene homolog 1 *	**YA > MA**	0.9783	**↑ miR-106a**
YWHAB	*tyrosine 3-monooxygenase/tryptophan 5-monooxygenase activation protein*, *beta polypeptide *	**YA > MA**	0.9493	**↑ miR-18b; 106a**
CDKN1B	*cyclin-dependent kinase inhibitor 1B (p27*, *Kip1)*	**YA > MA**	0.9014	**↑ miR-9**
SRC	*v-src sarcoma (Schmidt-Ruppin A-2) viral oncogene homolog (avian)*	**YA > MA**	0.9014	**↑ miR-9**
LCK	*LCK proto-oncogene*, *Src family tyrosine kinase *	**YA > MA**	0.8986	**↑ miR-18b**
RPS6KB1	*ribosomal protein S6 kinase*, *70kDa*, *polypeptide 1 *	**YA > MA**	0.8812	**↑ miR-33b**
STMN1	*stathmin 1 *	**YA > MA**	0.8812	**↑ hsa-9; 106a; miR-210**
YWHAZ	*tyrosine 3-monooxygenase/tryptophan 5-monooxygenase activation protein*, *zeta polypeptide *	**YA > MA**	0.8341	**↓ hsa-miR-372; ↑ miR-106a/b**
BCL2L1	*BCL2-like 1 *	**YA > MA**	0.8043	**↑ hsa-miR-9; miR-106a/b**
NRG1	*neuregulin 1 *	**YA > MA**	0.8043	**↑ hsa-miR-18b**
PARP1	*poly (ADP-ribose) polymerase 1*	**YA > MA**	0.8043	**↓ hsa-miR-372; ↑ hsa-miR-9; 33b; 106a/b**

Over expression of proteins in YA-BC group (YA > MA); under expression of proteins in YA-BC group (YA < MA).

**Fig 2:**
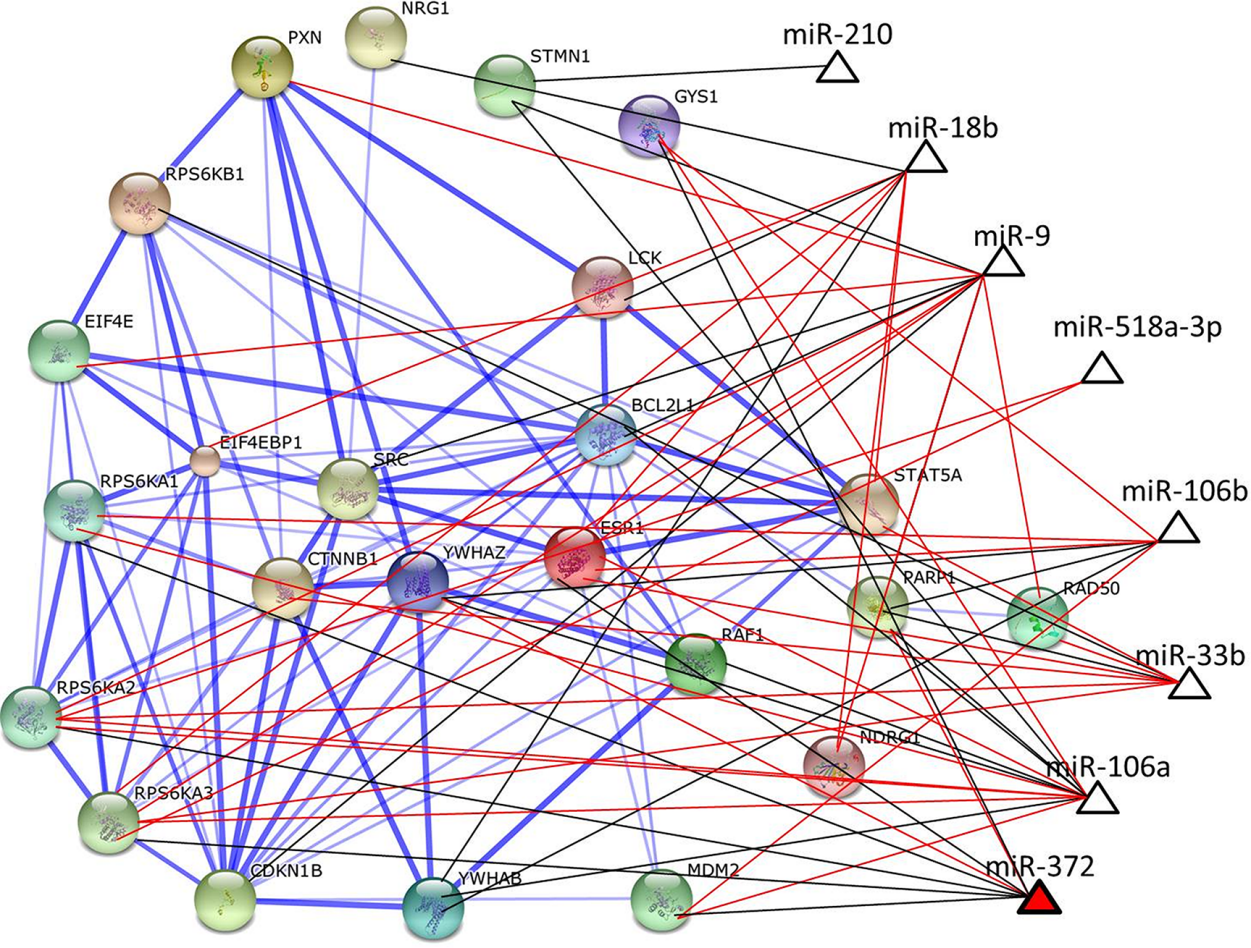
Protein-protein interaction and miRs network. This network represent over and under expression miRs (not fulfilled and fulfilled triangle respectively) in the YA-BC tumors interacting with their protein targets (circles). The inversely or coordinately regulation of proteins by miRs were represented by red or black edges respectively. The strong, or less strong protein-protein interactions based on databases, were represented by thicker or thinner blue edges respectively.

To better understand the interaction of eight miRs and their protein targets, we have performed a PPI network. Twenty-one out of the 24 differentially expressed proteins of the two groups were found in the human interactome database, and showed strong or less strong protein-protein interaction based on database reports (thick or thin blue edges, respectively in **Fig 2**). Therefore, STMN1, GYS1 and NDRG1 proteins were not interconnected to other proteins. In the PPI five more interconnected nodes were CDKN1B (14 connections), BCL2L1 (13 connections), SRC (11 connections), ESR1 (10 connections) and CTNNB1 (10 connections). The miR-proteins may follow inversely or coordinately regulation by miRs (black and red edges respectively in **Fig 2**). The biological processes outlined in GO analysis from the 21 interconnected proteins were listed in **S3 Table** and the more statistically (P≤0.05) significant biological processes associated with this network were: enzyme linked receptor protein signaling, neurotrophin signaling, cell cycle, regulation of apoptosis, metabolism regulation, development, proliferation and response to stress.

**Fig 3:**
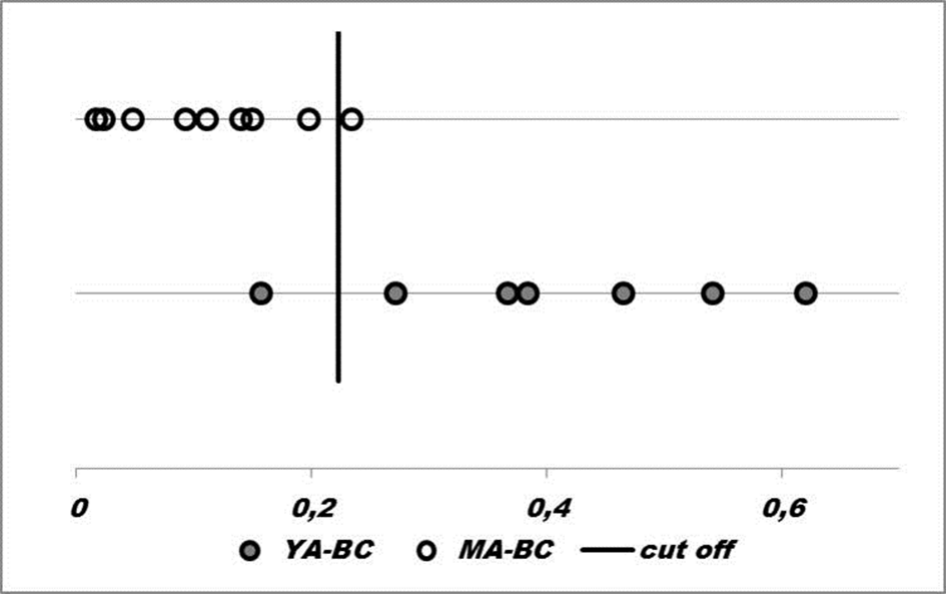
Samples classification based on proteins. Four index pairs of proteins classified samples (circles) into the right side of the cut-off mark corresponding to YA-BC group, and to the left side corresponding to MA-BC group.

Among the 24 proteins regulated by miRs, four index pairs encompassed by eight proteins (RAF1, BCL2L1, EIF4E, STAT5A, PARP1, ESR1, RPS6KA1 and YWHAZ) were chosen for sample classification. Individual samples were correctly classified with 95% of confidence to be assigned to the YA-BC group and with 89% to be assigned to the MA-BC group (**Fig 3**), showing that the four proteins index pairs differ between the two groups. In addition, these results suggested that RAF1, BCL2L1, EIF4E, STAT5A, PARP1, ESR1, RPS6KA1 and YWHAZ proteins discriminate YA-BC from MA-BC.

### Association between clinicopathological characteristics and proteins expression

The increased level of RAF1 protein expression and lower level of STAT5 protein expression were statistically associated (p = 0.02 and p = 0.002, respectively) with high advanced TNM. The increased level of PARP1 expression and lower level of RPS6KA1 expression were statistically associated (p = 0.03 and p = 0.03, respectively) with positive lymphonodes. A significant relationship (p = 0.03) was found between the lower level of EIF4E expression and high histological grade. The lower level of RPS6KA1 expression was statistically associated (p = 0.02) to larger tumor sizes (**S4 Table**).

### Association between proteins and mRNAs expression levels

We examined the regulatory effects of miRNAs on genes and proteins targets by verifying the concordance between mRNA and protein expression levels, obtained by microarray and RPPA assays respectively. Comparing the list of 24 proteins differentially expressed between YA-BC and MA-BC with the list of target genes differentially expressed between the two groups we found that miR-9 and miR-106a/b all over-expressed in YA-BC could reduce mRNA and proteins levels of ESR1 and RPS6KA1. Moreover, increased expression of YWHAZ mRNA and protein was observed, and it could happen due to the under-expression of miR-372 in YA-BC (**Table 5**). The mRNAs and proteins expression levels of EIF4E, PARP1 and BCL2L1 proteins were not concordant. The *RAF1* and *STAT5* mRNAs did not showed confidence statement over 80% (**Table 5**). The mRNA of *PARP1* did not reach the signal expression level higher that of the background, however qPCR analyses confirmed its under-expression in the YA-BC. In summary, ESR1, YWHAZ and RPS6KA1, exhibited concordant mRNAs and proteins expression levels with high confidence.

**Table 5:** Association between proteins and mRNA expression levels.

PROTEIN (RPPA)	Statement Protein	Confidence protein	mRNA (microarray)	Statement gene	Confidence genes
YWHAZ	YA > MA	0.834	YWHAZ	YA > MA	0.917
BCL2L1	YA > MA	0.804	BCL2L1	YA < MA	0.285
RAF1	YA > MA	0.978	RAF1	YA > MA	0.571
EIF4E	YA < MA	0.978	EIF4E	YA > MA	0.548
ESR1	YA < MA	0.899	ESR1	YA < MA	0.917
PARP1	YA > MA	0.804	PARP1*	YA < MA*	*
RPS6KA1	YA < MA	0.881	RPS6KA1	YA < MA	0.833
STAT5A	YA < MA	0.908	STAT5A	YA < MA	0.548

Over expression of proteins in YA-BC group (YA > MA); under expression of proteins in YA-BC group (YA < MA).

* Performed by qPCR.

### Gene expression profile from fibroblast-enriched stroma

Determination of microRNA profiles was not possible in samples obtained by LCM because of sample size limitations and little amount of material recovered. However, the mRNA expression profile of fibroblast-enriched stroma showed 346 genes differentially expressed between YA-BC and MA-BC. A comparative analysis of these genes with the 1306 genes differentially expressed in matched whole tumors resulted in 38 genes within the intersection between the two groups and 308 exclusively regulated genes in the microdissected stroma tissue, with 154 up-regulated genes and 154 down-regulated genes in YA-BC compared to MA-BC tumors. These genes were classified in different biological categories, including metabolism, glycosphingolipid, focal adhesion, metabolism of xenobiotics, cancer and cytokine-cytokine interaction **(S5 Table).**

Integration of the exclusively regulated 308 genes in the stroma with miRNA target prediction indicated 129 stromal genes that may be regulated by at least one of the eight miRNAs differentially expressed between YA-BC and MA-BC. Eighty-seven out of 129 genes were predicted to be inversely regulated by these miRNAs.

**Fig 4:**
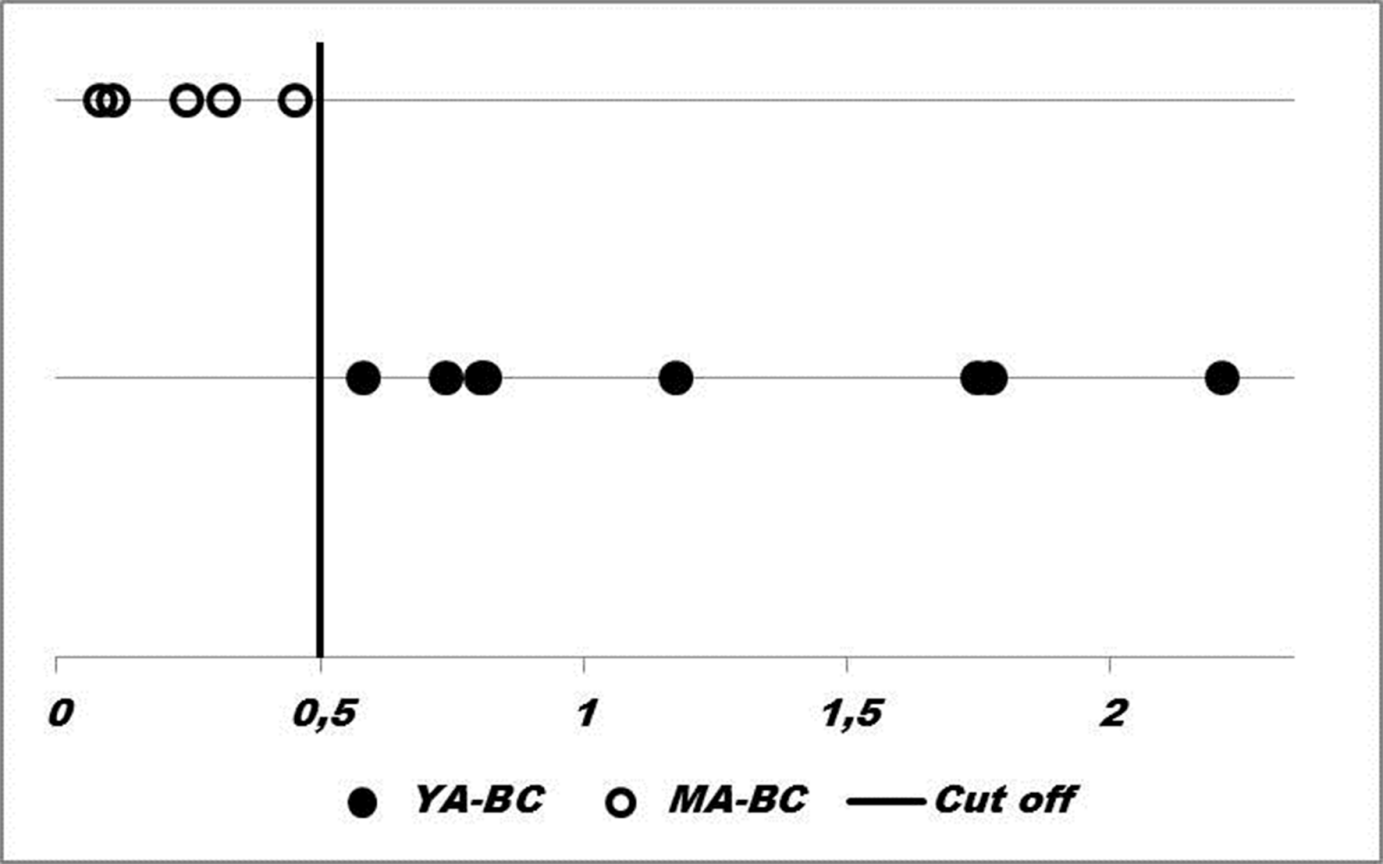
Samples classification based on stromal genes. Four index pairs of stromal genes classified samples (circles) into the right side of cut-off mark corresponding to YA-BC group, and to the left side corresponding to MA-BC group.

To perform sample classification we selected four index pairs comprised of eight stromal genes regulated by miRs, and related to significantly enriched pathways. To simulate classification, the index pairs included up regulation of *UQCRQ*, *ALDH1A3*, *EGLN1*, *IGF1*, and down regulation of *FUT9*, *IDI2*, *PDHX*, *and CCL18* in YA-BC. Individual samples were correctly classified with 100% accuracy for both groups (**Fig 4**). Thus, an index including eight stromal genes discriminated tumor microenvironments between the two groups.

## Discussion

Previously clinicopathological studies of Brazilian patients with diagnosed BC under 35 years, highlighted a prevalence of cases exhibiting invasive ductal carcinomas, intermediate or high histological grade and hormone receptors positivity [**14, 47, 48**]. In the present evaluation of a group of young Brazilian patients as compared to older counterparts, we confirmed these previous results related to aggressive patterns associated to advanced tumor stage and grade as well as to the large size of the lesions.

In the current study we identified eight differentially expressed miRs with seven over-expressed in tumors of YA-BC and, only one miR (miR-372) was under-expressed. Most of these miRs were previously implicated in regulating pathways involved with BC malignancy. The over expression of miR-9, miR-210, miR-106b, miR-33b, miR-18b was formerly related to poor prognosis, suggesting those miRs as aggressive biomarkers [**49–53**]. The under-expression of miR-372 was related to metastatic tumors, suggesting reduction of miR-372 expression as a poor prognosis biomarker [**54**]. Little is known about the contribution of miR-518a-3p in breast carcinoma, although our results showed significant association between advanced TNM status and overexpression levels of miR-518a-3p, suggesting that this miR could influence the aggressive behavior of YA-BC. Combined miRs and mRNAs differentially expressed between tumors of younger and middle age groups indicated some target genes associated to proliferation in the former group. Recently, Penã-Chilet et al [**24**] performed a comprehensive study of miR expression profiling using Affymetrix2.0 array on paraffin-embedded tumor tissues of breast cancer patients aged 35 years or younger and patients older than 45 years. Differences in the expression of six miRs, and in silico enrichment analyses resulted in predictive targets involved with proliferation pathways, cell adhesion, apoptosis, extracellular matrix and cell motility. Although, the miRs differentially expressed in the present study did not overlap with the miRs mentioned in the above report, but our results also highlighted the importance of proliferation processes related to YA-BC based on miRs expression. In addition, the divergence of miRs profiles may be due to ethnic differences, since the genetic background of the Brazilian population is heterogeneous.

The role of miRs regulation in mRNA expression emphasized the importance of four over-expressed genes (*YWHAZ*, *DUSP2*, *DUSP8* and *PIGS*), and four under-expressed genes (*ESR1*, *RPS6KA1*, *BCL7 and PARP12)* in the YA-BC group. Literature search confirmed that the low expression level of *BCL7* target gene was previously related to tumor progression and development in carcinomas [**55**]. The role of *DUSP2* and *DUSP8* genes coding phosphatases regulating MAPK signaling pathway confirmed the contribution of these target genes to the development and progression of many cancers [**56,57**]. In addition, PARP12, a mono-ADP-ribose polymerase (PARPs), is an interferon induced gene recruited to stress granules after exposure to oxidative stress. PARP12 was involved in control of protein translation and inflammation [**58**]. Its decreased level in tumors of YA-BC patients as compared to MA-BC may impair the response to great oxidant stress previously reported in early onset breast cancer [**59**]. PIGS protein is one of the five subunits of the glycosylphosphatidil inositol transamidase complex (GPIT) important to attach GPI (glycosylphosphatidil inositol anchor) to proteins. Elevated GPI-anchored proteins expression levels has been associated with mesenchymal stem cells which promote tumor cell growth in breast cancer [**60**]. Decrease of *ESR1* in tumors of YA-BC patients as compared to MA-BC patients may cause tumor growth no longer under estrogen control [**12, 61**]. *RPS6KA1* (ribosomal protein S6 kinase or S6K1) is a major mTOR downstream signaling molecule. Phosphorylated S6K1 limits insulin/IGF-1 mediated PI3K activation generating a feedback signal loop by phosphorylation of TOR repressor domain with attenuation of mTOR ability in protein synthesis [**62, 63**]. Decreased levels of S6K1 in young patients tumors may result in maintaining mTOR activation. Due to these evidences, our results raised the hypothesis that changes in *ERS1*, *RPS6KA1*, *YWHAZ*, *BCL7*, *PARP12*, *DUSP2*, *DUSP8* and *PIGS* target genes may contribute to the aggressive behavior of YA-BC tumors.

Besides direct action on the UTRs of target genes, miRs may repress at the translational and protein transduction level [**28**]. Many miRs stimulate secondary effects through other proteins, not only altering the expression levels of target proteins but also the complex network associated with targets [**29**]. Due to this complex mechanism of miRs regulation, to approach protein expression profile has gained importance for the comprehension of the biological process in breast cancer. Results of other reports have supported that the structure and dynamics of protein networks are disturbed in complex diseases such as cancer [**64**]. Our results indicate that 21 of the 24 differentially expressed proteins were interconnected, suggesting that miRs may co-regulate proteins with similar functional categories showed by mRNA expression profile. The over expression of BCL2L1, PARP1, RAF1 proteins [**65, 66**] and under expression of ESR1, EIF4E, STAT5A, RPS6KA1 proteins [**67–72**] were previously associated with tumors aggressive behavior indicating that they may be considered as potential biomarkers of BC aggressiveness. In addition, the identification of BCL2L1 and ESR1 as network nodes emphasized the importance of both proteins for the acquisition of aggressive characteristics. Little is known about the influence of YWHAZ protein on breast cancer aggressiveness but the interaction of miR-372 with YWHAZ gene and protein was previously described in embryonic cell differentiation [**73**]. The concordant changes in expression levels of mRNAs and proteins of ESR1, YWHAZ and RPS6KA1 suggested interactions with classic regulatory effect by miRs. The remaining mRNA/proteins interactions did not follow the classic regulation by miRs. Therefore, RPPA methodology seems to provide different information from microarray analysis [**28**].

Previous studies have identified stromal gene signatures relevant for breast cancer progression [**74, 75**]. Tichy et al [**1**] suggested that the difference between breast carcinoma of young patients and those of older patients could be partly due to the microenvironment that involved malignant epithelial cells. Our study focused on differentially gene expression profiles between tumors form stroma enriched with fibroblasts of YA and MA. Our results revealed distinct gene expression profiles that after *in silico* analyses indicated enrichment for pathways such as metabolism, focal adhesion, and cytokine-cytokine interaction. It is noteworthy that some of the listed genes were previously described as differentially expressed in carcinoma-associated fibroblasts as compared to normal fibroblasts in human breast cancer [**76, 77**]. A novel contribution of our study was to identify the combination of eight stromal genes that could distinguish tumors in YA-BC and MA-BC, suggesting distinct biological characteristics in the stromal component between both groups of patients. In addition, our results suggested over-expression of *UQCRQ*, *ALDH1A3*, *EGLN1*, *IGF1*, *and under-expression of FUT9*, *IDI2*, *PDHX* and *CCL18* as potential stromal genes that could contribute to aggressive behavior in YA-BC. AldH1A3 (aldehyde dehydrogenase family 1, subfamily A3) activity has been used to identify stem-like cells within the tumor epithelial cells [**78**]. The increasing expression of PDHX in late onset tumors, which inhibits the conversion of pyruvate to acetyl CoA in the citric acid cycle may lead to pyruvate, lactate accumulation and over consumption of glucose in the stromal compartment of older patients tumors [**79**]. We noted in MA-BC tumors the increase in metabolic markers which includes proteins involved in glycosylation in the Golgi such as FUT9. The enzyme encoded by FUT9 gene is the earliest alfa-3-fucosyl transferase expressed during the first two months of embyogenesis and it is also important for cell-cell interactions [**80**]. CCL18 is a chemokine C-C motif ligand that is up-regulated in breast cancer determining the severity of breast cancer malignancy [**81**]. The protein encoded by *EGLN1* gene catalyzes the post-translational formation of 4-hydroxyproline into hypoxia-inducible factor (HIF) alpha proteins, and it plays a central role in mammalian oxygen homeostasis [**82**]. The protein encoded by *IGF1* gene is similar to insulin, in function and structure and belong to a family of proteins involved in mediating growth and development of breast cancer [**83**]. The *UQCRQ* gene encodes a ubiquinone-binding protein of low molecular mass which is part of the mitochondrial respiratory chain [**84**].

We employed an integrative analysis, a systems biology approach, which represents a comprehensive method to understand the flow of biological information, underling complex biological traits [**85**]. In the present work we showed that the combination of microRNA screening, transcriptome and proteomic profiles could identify biological differences between early and late onset ER positive BC which suggest aggressive behavior biomarkers that may represent new insights to the treatment of estrogen positive YA-BC.

## Conclusion

In conclusion, in this study we identified signatures that discriminate ER positive tumors between YA-BC and MA-BC combining microRNA screening, protein and gene analysis. Moreover, this integrated analysis uncovered a set of miRs/genes/proteins that could be mediators of biological characteristics involved with tumor aggressiveness of young patients. However, a larger sample size must be used to confirm our results.

## Supporting Information

S1 Table Differentially expressed mRNAs (YA-BC *vs* MA-BC). (DOC)

S2 Table Association between mRNAs and tumors clinicopathological characteristics. (DOC)

S3 Table Biological processes associated with 21 interconnected protein. (DOC)

S4 Table Association between proteins and tumors clinicopathological characteristics. (DOC)

S5 Table Biological stromal genes functions of of YA-BC and MA-BC tumors microenvironments (*Webgestalt*). (DOC)
