# Supplementary material for: A Set of miRNAs, Their Gene and Protein Targets and Stromal Genes Distinguish Early from Late Onset ER Positive Breast Cancer
Source: PLoS One. 2016 May 6;11(5):e0154325. doi: 10.1371/journal.pone.0154325 (PMC4859528; doi:10.1371/journal.pone.0154325)
Supplement: S1 Table — (DOC) [file pone.0154325.s001.doc]

**S1 Table.** Differentially expressed mRNAs (YA-BC *vs* MA-BC)

|  | |  |  |
| --- | --- | --- | --- |
| **GeneSymbol** | **Statement** | **miR** | **Number of overlapping prediction databases** |
| AACS | YA < MA | hsa-miR-33b | 5 |
|  |  | hsa-miR-33b* | 3 |
| AASDHPPT | YA < MA | hsa-miR-33b | 3 |
|  |  | hsa-miR-106a | 3 |
|  |  | hsa-miR-106b | 3 |
| ABCB8 | YA > MA | hsa-miR-9 | 5 |
|  |  | hsa-miR-33b | 4 |
|  |  | hsa-miR-18b | 4 |
| ABCC5 | YA < MA | hsa-miR-9 | 3 |
|  |  | hsa-miR-372 | 5 |
|  |  | hsa-miR-210 | 3 |
|  |  | hsa-miR-106a | 7 |
|  |  | hsa-miR-106a* | 3 |
|  |  | hsa-miR-106b | 5 |
|  |  | hsa-miR-18b | 4 |
| ABCC9 | YA < MA | hsa-miR-33b | 3 |
|  |  | hsa-miR-106a | 3 |
|  |  | hsa-miR-18b | 4 |
| ABCD1 | YA > MA | hsa-miR-9 | 6 |
|  |  | hsa-miR-210 | 5 |
| ABHD4 | YA > MA | hsa-miR-106a | 4 |
|  |  | hsa-miR-18b | 3 |
| ABHD9 | YA < MA | hsa-miR-518a-3p | 5 |
| ABL1 | YA > MA | hsa-miR-210 | 3 |
|  |  | hsa-miR-106a | 3 |
|  |  | hsa-miR-18b | 4 |
| ACIN1 | YA > MA | hsa-miR-106a | 3 |
|  |  | hsa-miR-106b | 5 |
| ACTB | YA > MA | hsa-miR-33b | 3 |
|  |  | hsa-miR-18b | 4 |
| ACTR2 | YA < MA | hsa-miR-518a-3p | 5 |
|  |  | hsa-miR-106a | 3 |
|  |  | hsa-miR-106b | 5 |
| ADIPOR2 | YA < MA | hsa-miR-372 | 5 |
|  |  | hsa-miR-106a | 5 |
|  |  | hsa-miR-106b | 6 |
| AFF3 | YA < MA | hsa-miR-33b | 4 |
|  |  | hsa-miR-33b* | 3 |
|  |  | hsa-miR-106a | 4 |
| AHCY | YA > MA | hsa-miR-106a | 3 |
|  |  | hsa-miR-106b | 5 |
| AHCYL1 | YA > MA | hsa-miR-372 | 3 |
|  |  | hsa-miR-33b | 3 |
|  |  | hsa-miR-106a | 4 |
|  |  | hsa-miR-18b | 3 |
| AIP | YA > MA | hsa-miR-372 | 3 |
| AKAP6 | YA < MA | hsa-miR-33b | 5 |
|  |  | hsa-miR-33b* | 3 |
|  |  | hsa-miR-106a | 5 |
|  |  | hsa-miR-106a* | 3 |
|  |  | hsa-miR-106b | 6 |
| ALDH1A2 | YA > MA | hsa-miR-33b | 3 |
|  |  | hsa-miR-18b | 6 |
| ALPK1 | YA < MA | hsa-miR-106a | 3 |
|  |  | hsa-miR-106b | 4 |
| ANGEL2 | YA < MA | hsa-miR-372 | 5 |
|  |  | hsa-miR-33b | 3 |
|  |  | hsa-miR-106a | 5 |
|  |  | hsa-miR-106b | 6 |
|  |  | hsa-miR-18b | 5 |
| ANKFY1 | YA < MA | hsa-miR-9 | 5 |
|  |  | hsa-miR-372 | 6 |
|  |  | hsa-miR-33b | 4 |
|  |  | hsa-miR-106a | 6 |
|  |  | hsa-miR-106a* | 3 |
|  |  | hsa-miR-106b | 8 |
|  |  | hsa-miR-106b* | 3 |
|  |  | hsa-miR-18b | 6 |
| ANKRD17 | YA < MA | hsa-miR-106a | 4 |
| ANKRD34A | YA < MA | hsa-miR-106a | 3 |
| ANKRD7 | YA < MA | hsa-miR-18b | 3 |
| ANLN | YA > MA | hsa-miR-106a | 3 |
|  |  | hsa-miR-106b | 3 |
|  |  | hsa-miR-18b | 4 |
| ANXA5 | YA > MA | hsa-miR-18b | 4 |
| AP3M2 | YA < MA | hsa-miR-372 | 3 |
| AP3S2 | YA > MA | hsa-miR-18b | 5 |
| AP4E1 | YA < MA | hsa-miR-9 | 7 |
|  |  | hsa-miR-33b | 3 |
|  |  | hsa-miR-106a | 5 |
|  |  | hsa-miR-106a* | 3 |
|  |  | hsa-miR-106b | 3 |
| APH1B | YA < MA | hsa-miR-372 | 4 |
| ARHGAP19 | YA < MA | hsa-miR-9 | 5 |
|  |  | hsa-miR-106a | 4 |
|  |  | hsa-miR-106a* | 3 |
| ARID1B | YA < MA | hsa-miR-9 | 6 |
|  |  | hsa-miR-106a | 5 |
|  |  | hsa-miR-106b | 3 |
| ARL1 | YA < MA | hsa-miR-9 | 5 |
|  |  | hsa-miR-372 | 5 |
|  |  | hsa-miR-106a | 6 |
|  |  | hsa-miR-106b | 6 |
| ARMCX5 | YA < MA | hsa-miR-33b | 5 |
| ARSG | YA < MA | hsa-miR-518a-3p | 3 |
| ASCC2 | YA > MA | hsa-miR-18b | 5 |
| ASPHD2 | YA > MA | hsa-miR-9 | 3 |
| ATF3 | YA < MA | hsa-miR-9 | 5 |
|  |  | hsa-miR-518a-3p | 3 |
| ATF7IP2 | YA < MA | hsa-miR-372 | 5 |
|  |  | hsa-miR-106a | 5 |
|  |  | hsa-miR-106b | 5 |
| ATP6V1G1 | YA < MA | hsa-miR-106a | 4 |
| ATP8B4 | YA < MA | hsa-miR-106a | 4 |
| ATXN2L | YA > MA | hsa-miR-18b | 5 |
| B3GNT7 | YA < MA | hsa-miR-33b | 3 |
| BAALC | YA < MA | hsa-miR-33b | 5 |
|  |  | hsa-miR-33b* | 3 |
|  |  | hsa-miR-106a | 5 |
|  |  | hsa-miR-106a* | 3 |
|  |  | hsa-miR-106b | 5 |
|  |  | hsa-miR-18b | 5 |
| BACE1 | YA < MA | hsa-miR-9 | 6 |
|  |  | hsa-miR-372 | 3 |
|  |  | hsa-miR-106a | 4 |
|  |  | hsa-miR-106a* | 3 |
|  |  | hsa-miR-106b | 3 |
| BAG4 | YA < MA | hsa-miR-9 | 6 |
| BCAS1 | YA > MA | hsa-miR-372 | 5 |
|  |  | hsa-miR-106a | 5 |
|  |  | hsa-miR-106a* | 3 |
|  |  | hsa-miR-106b | 5 |
| BCL2 | #N/D | hsa-miR-9 | 3 |
|  |  | hsa-miR-518a-3p | 4 |
|  |  | hsa-miR-372 | 5 |
|  |  | hsa-miR-33b | 3 |
|  |  | hsa-miR-106a | 6 |
|  |  | hsa-miR-106b | 5 |
|  |  | hsa-miR-106b* | 3 |
| BCL2L1 | #N/D | hsa-miR-9 | 3 |
|  |  | hsa-miR-106a | 4 |
| BCL7A | YA < MA | hsa-miR-518a-3p | 4 |
|  |  | hsa-miR-372 | 3 |
|  |  | hsa-miR-18b | 3 |
| BCOR | YA > MA | hsa-miR-33b | 5 |
|  |  | hsa-miR-33b* | 3 |
| BDNF | YA < MA | hsa-miR-210 | 3 |
| BET1L | YA > MA | hsa-miR-9 | 5 |
|  |  | hsa-miR-518a-3p | 3 |
|  |  | hsa-miR-106a | 3 |
|  |  | hsa-miR-106b | 3 |
|  |  | hsa-miR-18b | 3 |
| BMP3 | YA < MA | hsa-miR-9 | 4 |
|  |  | hsa-miR-518a-3p | 3 |
|  |  | hsa-miR-372 | 3 |
|  |  | hsa-miR-33b | 5 |
|  |  | hsa-miR-106a | 4 |
|  |  | hsa-miR-106b | 4 |
| BPHL | YA > MA | hsa-miR-372 | 5 |
|  |  | hsa-miR-106a | 5 |
|  |  | hsa-miR-106b | 5 |
| BRCA1 | YA < MA | hsa-miR-9 | 4 |
|  |  | hsa-miR-372 | 4 |
|  |  | hsa-miR-106a | 4 |
|  |  | hsa-miR-106b | 4 |
| BTN2A1 | YA < MA | hsa-miR-9 | 5 |
|  |  | hsa-miR-210 | 3 |
| C10orf118 | YA < MA | hsa-miR-9 | 5 |
|  |  | hsa-miR-372 | 5 |
|  |  | hsa-miR-33b | 4 |
|  |  | hsa-miR-106a | 5 |
|  |  | hsa-miR-106a* | 3 |
|  |  | hsa-miR-106b | 5 |
| C10orf140 | YA < MA | hsa-miR-9 | 3 |
|  |  | hsa-miR-372 | 3 |
|  |  | hsa-miR-33b | 4 |
|  |  | hsa-miR-33b* | 3 |
|  |  | hsa-miR-106a | 3 |
|  |  | hsa-miR-106b | 5 |
| C10orf76 | YA < MA | hsa-miR-372 | 5 |
|  |  | hsa-miR-33b | 3 |
|  |  | hsa-miR-106a | 5 |
|  |  | hsa-miR-106b | 5 |
|  |  | hsa-miR-18b | 5 |
| C11orf54 | YA < MA | hsa-miR-9 | 5 |
|  |  | hsa-miR-372 | 5 |
|  |  | hsa-miR-106a | 5 |
|  |  | hsa-miR-106b | 5 |
| C12orf41 | YA < MA | hsa-miR-33b | 3 |
| C14orf145 | YA > MA | hsa-miR-372 | 5 |
|  |  | hsa-miR-106a | 4 |
|  |  | hsa-miR-106b | 5 |
| C19orf55 | YA < MA | hsa-miR-9 | 5 |
|  |  | hsa-miR-372 | 5 |
|  |  | hsa-miR-106a | 5 |
|  |  | hsa-miR-106b | 5 |
|  |  | hsa-miR-18b | 5 |
| C1orf128 | YA > MA | hsa-miR-210 | 5 |
| C1orf27 | YA < MA | hsa-miR-33b | 3 |
| C1orf43 | YA > MA | hsa-miR-518a-3p | 6 |
|  |  | hsa-miR-33b | 5 |
|  |  | hsa-miR-18b | 3 |
| C1orf9 | YA < MA | hsa-miR-372 | 5 |
|  |  | hsa-miR-106a | 5 |
|  |  | hsa-miR-106b | 5 |
|  |  | hsa-miR-18b | 7 |
|  |  | hsa-miR-18b* | 3 |
| C20orf4 | YA > MA | hsa-miR-9 | 5 |
| C2orf42 | YA < MA | hsa-miR-372 | 3 |
| C2orf63 | YA < MA | hsa-miR-106a | 4 |
|  |  | hsa-miR-106b | 3 |
|  |  | hsa-miR-18b | 5 |
|  |  | hsa-miR-18b* | 3 |
| C3orf52 | YA < MA | hsa-miR-372 | 4 |
|  |  | hsa-miR-106a | 5 |
|  |  | hsa-miR-106b | 5 |
|  |  | hsa-miR-106b* | 3 |
|  |  | hsa-miR-18b | 3 |
| C6orf182 | YA > MA | hsa-miR-106a | 3 |
| C6orf201 | YA < MA | hsa-miR-372 | 5 |
|  |  | hsa-miR-106a | 6 |
|  |  | hsa-miR-106b | 6 |
| C7orf45 | YA < MA | hsa-miR-9 | 5 |
|  |  | hsa-miR-372 | 3 |
|  |  | hsa-miR-106a | 4 |
|  |  | hsa-miR-106b | 4 |
| C7orf58 | YA > MA | hsa-miR-33b | 3 |
| C8orf4 | YA > MA | hsa-miR-372 | 3 |
| C9orf114 | YA > MA | hsa-miR-9 | 3 |
|  |  | hsa-miR-18b | 5 |
| C9orf78 | YA > MA | hsa-miR-372 | 5 |
|  |  | hsa-miR-106a | 5 |
|  |  | hsa-miR-106b | 5 |
|  |  | hsa-miR-18b | 5 |
| CA7 | YA < MA | hsa-miR-9 | 4 |
| CABP5 | YA < MA | hsa-miR-9 | 5 |
|  |  | hsa-miR-372 | 3 |
| CALN1 | YA < MA | hsa-miR-9 | 4 |
|  |  | hsa-miR-33b | 5 |
|  |  | hsa-miR-106a | 4 |
|  |  | hsa-miR-106b | 4 |
|  |  | hsa-miR-106b* | 3 |
| CAMK2G | YA < MA | hsa-miR-33b | 5 |
|  |  | hsa-miR-106a | 5 |
|  |  | hsa-miR-106a* | 3 |
|  |  | hsa-miR-18b | 3 |
| CAPZB | YA > MA | hsa-miR-33b | 3 |
|  |  | hsa-miR-210 | 3 |
| CASC1 | YA < MA | hsa-miR-372 | 3 |
| CASP7 | YA < MA | hsa-miR-372 | 4 |
|  |  | hsa-miR-106a | 6 |
|  |  | hsa-miR-106a* | 3 |
|  |  | hsa-miR-106b | 6 |
|  |  | hsa-miR-18b | 5 |
|  |  | hsa-miR-18b* | 3 |
| CCBE1 | YA < MA | hsa-miR-372 | 6 |
|  |  | hsa-miR-106a | 5 |
|  |  | hsa-miR-106b | 6 |
| CCDC126 | YA < MA | hsa-miR-9 | 6 |
|  |  | hsa-miR-18b | 3 |
| CCDC47 | YA > MA | hsa-miR-372 | 5 |
| CCDC80 | YA > MA | hsa-miR-106a | 5 |
|  |  | hsa-miR-106b | 4 |
| CCKBR | YA < MA | hsa-miR-210 | 4 |
| CCL11 | YA > MA | hsa-miR-33b | 5 |
| CCL13 | YA > MA | hsa-miR-33b | 3 |
| CCT6B | YA < MA | hsa-miR-106a | 3 |
| CD109 | YA < MA | hsa-miR-9 | 6 |
|  |  | hsa-miR-372 | 5 |
|  |  | hsa-miR-106a | 5 |
|  |  | hsa-miR-106a* | 3 |
|  |  | hsa-miR-106b | 5 |
| CD300LG | YA < MA | hsa-miR-372 | 5 |
|  |  | hsa-miR-18b | 5 |
| CD84 | YA > MA | hsa-miR-9 | 5 |
|  |  | hsa-miR-106a | 5 |
|  |  | hsa-miR-106b | 3 |
| CD93 | YA > MA | hsa-miR-9 | 4 |
|  |  | hsa-miR-372 | 5 |
|  |  | hsa-miR-33b | 5 |
|  |  | hsa-miR-33b* | 3 |
|  |  | hsa-miR-18b | 3 |
| CDC40 | YA > MA | hsa-miR-9 | 5 |
|  |  | hsa-miR-372 | 6 |
|  |  | hsa-miR-33b | 4 |
|  |  | hsa-miR-106a | 6 |
|  |  | hsa-miR-106b | 6 |
| CDCA7 | YA > MA | hsa-miR-372 | 7 |
|  |  | hsa-miR-106a | 7 |
|  |  | hsa-miR-106a* | 3 |
|  |  | hsa-miR-106b | 6 |
| CDCP1 | YA > MA | hsa-miR-9 | 4 |
|  |  | hsa-miR-33b | 4 |
|  |  | hsa-miR-33b* | 3 |
|  |  | hsa-miR-210 | 6 |
|  |  | hsa-miR-18b | 5 |
| CDK2 | YA > MA | hsa-miR-372 | 3 |
|  |  | hsa-miR-106a | 5 |
|  |  | hsa-miR-106b | 3 |
|  |  | hsa-miR-18b | 5 |
| CDON | YA < MA | hsa-miR-9 | 3 |
|  |  | hsa-miR-33b | 5 |
|  |  | hsa-miR-33b* | 3 |
|  |  | hsa-miR-210 | 5 |
| CEACAM7 | YA > MA | hsa-miR-33b | 4 |
| CENPO | YA > MA | hsa-miR-372 | 5 |
|  |  | hsa-miR-106a | 5 |
|  |  | hsa-miR-106b | 6 |
| CHODL | YA < MA | hsa-miR-33b | 4 |
| CHP | YA < MA | hsa-miR-18b | 3 |
| CHST3 | YA > MA | hsa-miR-9 | 3 |
|  |  | hsa-miR-372 | 5 |
| CIAPIN1 | YA > MA | hsa-miR-210 | 5 |
|  |  | hsa-miR-106a | 3 |
|  |  | hsa-miR-106b | 5 |
| CNGB3 | YA < MA | hsa-miR-9 | 5 |
|  |  | hsa-miR-372 | 5 |
|  |  | hsa-miR-33b | 5 |
|  |  | hsa-miR-106a | 5 |
|  |  | hsa-miR-106a* | 3 |
|  |  | hsa-miR-106b | 5 |
| CNNM4 | YA < MA | hsa-miR-9 | 5 |
|  |  | hsa-miR-106a | 4 |
| COL4A1 | YA > MA | hsa-miR-9 | 4 |
|  |  | hsa-miR-372 | 3 |
|  |  | hsa-miR-33b | 4 |
|  |  | hsa-miR-106a | 4 |
|  |  | hsa-miR-106b | 4 |
| COMT | YA > MA | hsa-miR-33b | 3 |
| CRB1 | YA < MA | hsa-miR-18b | 5 |
| CRB2 | YA > MA | hsa-miR-106a | 4 |
| CREB3L3 | YA > MA | hsa-miR-210 | 5 |
| CSDA | YA > MA | hsa-miR-9 | 8 |
|  |  | hsa-miR-18b | 4 |
| CSNK1A1 | YA > MA | hsa-miR-9 | 6 |
| CTAGE1 | YA > MA | hsa-miR-106a | 4 |
|  |  | hsa-miR-106b | 4 |
| CTDSPL2 | YA < MA | hsa-miR-372 | 5 |
|  |  | hsa-miR-33b | 5 |
|  |  | hsa-miR-106a | 5 |
|  |  | hsa-miR-106b | 7 |
|  |  | hsa-miR-106b* | 3 |
| CTNNBL1 | YA > MA | hsa-miR-18b | 3 |
| CUGBP2 | YA > MA | hsa-miR-9 | 5 |
|  |  | hsa-miR-372 | 6 |
|  |  | hsa-miR-33b | 5 |
|  |  | hsa-miR-210 | 5 |
|  |  | hsa-miR-106a | 5 |
|  |  | hsa-miR-106b | 5 |
| CXCL17 | YA < MA | hsa-miR-33b | 3 |
| CXorf38 | YA < MA | hsa-miR-372 | 5 |
|  |  | hsa-miR-106a | 5 |
|  |  | hsa-miR-106b | 5 |
| CYP19A1 | YA < MA | hsa-miR-518a-3p | 3 |
|  |  | hsa-miR-106a | 4 |
|  |  | hsa-miR-106b | 4 |
|  |  | hsa-miR-18b | 3 |
|  |  | hsa-miR-18b* | 3 |
| CYP4A11 | YA > MA | hsa-miR-33b | 3 |
|  |  | hsa-miR-18b | 3 |
| DAZAP1 | YA < MA | hsa-miR-210 | 4 |
| DCC | YA < MA | hsa-miR-9 | 5 |
|  |  | hsa-miR-33b | 3 |
| DCTN6 | YA > MA | hsa-miR-106a | 3 |
|  |  | hsa-miR-106b | 5 |
| DDX24 | YA > MA | hsa-miR-33b | 5 |
| DDX59 | YA > MA | hsa-miR-372 | 3 |
|  |  | hsa-miR-106a | 3 |
|  |  | hsa-miR-106b | 3 |
| DDX6 | YA > MA | hsa-miR-9 | 5 |
|  |  | hsa-miR-372 | 3 |
|  |  | hsa-miR-33b | 3 |
|  |  | hsa-miR-18b | 4 |
| DGUOK | YA < MA | hsa-miR-18b | 3 |
| DHCR24 | YA < MA | hsa-miR-33b | 3 |
|  |  | hsa-miR-33b* | 3 |
| DKK2 | YA > MA | hsa-miR-9 | 6 |
|  |  | hsa-miR-33b | 3 |
|  |  | hsa-miR-106a | 5 |
|  |  | hsa-miR-106a* | 3 |
|  |  | hsa-miR-106b | 5 |
| DKK3 | YA > MA | hsa-miR-33b | 3 |
| DLC1 | YA > MA | hsa-miR-518a-3p | 3 |
|  |  | hsa-miR-106a | 5 |
|  |  | hsa-miR-106b | 7 |
|  |  | hsa-miR-18b | 3 |
| DLX1 | YA > MA | hsa-miR-210 | 5 |
| DNAH5 | YA < MA | hsa-miR-33b | 3 |
| DNAJB12 | YA < MA | hsa-miR-9 | 6 |
|  |  | hsa-miR-372 | 5 |
| DNAJB14 | YA < MA | hsa-miR-9 | 4 |
| DNM1L | YA < MA | hsa-miR-372 | 5 |
|  |  | hsa-miR-33b | 4 |
|  |  | hsa-miR-106a | 3 |
|  |  | hsa-miR-106b | 3 |
| DNM3 | YA < MA | hsa-miR-9 | 3 |
|  |  | hsa-miR-372 | 5 |
|  |  | hsa-miR-106a | 4 |
|  |  | hsa-miR-106b | 4 |
| DNMT3A | YA < MA | hsa-miR-106b | 4 |
| DOLPP1 | YA < MA | hsa-miR-9 | 5 |
|  |  | hsa-miR-518a-3p | 4 |
| DPM2 | YA > MA | hsa-miR-9 | 3 |
| DPP3 | YA > MA | hsa-miR-372 | 4 |
|  |  | hsa-miR-106a | 4 |
|  |  | hsa-miR-106b | 4 |
| DRD1 | YA < MA | hsa-miR-372 | 6 |
|  |  | hsa-miR-33b | 3 |
|  |  | hsa-miR-106a | 6 |
|  |  | hsa-miR-106b | 6 |
| DSC2 | YA > MA | hsa-miR-33b | 5 |
|  |  | hsa-miR-33b* | 3 |
| DSE | YA > MA | hsa-miR-9 | 5 |
| DSEL | YA > MA | hsa-miR-372 | 4 |
|  |  | hsa-miR-33b | 4 |
|  |  | hsa-miR-106a | 3 |
| DST | YA < MA | hsa-miR-372 | 3 |
| DUSP2 | YA > MA | hsa-miR-372 | 8 |
|  |  | hsa-miR-106b | 8 |
| DUSP3 | YA > MA | hsa-miR-372 | 3 |
|  |  | hsa-miR-33b | 4 |
|  |  | hsa-miR-33b* | 3 |
|  |  | hsa-miR-106a | 6 |
|  |  | hsa-miR-106a* | 3 |
|  |  | hsa-miR-106b | 3 |
|  |  | hsa-miR-18b | 5 |
|  |  | hsa-miR-18b* | 3 |
| DUSP8 | YA < MA | hsa-miR-9 | 4 |
|  |  | hsa-miR-372 | 6 |
|  |  | hsa-miR-33b | 3 |
|  |  | hsa-miR-106a | 6 |
|  |  | hsa-miR-106a* | 3 |
|  |  | hsa-miR-106b | 8 |
|  |  | hsa-miR-106b* | 5 |
|  |  | hsa-miR-18b | 4 |
| DZIP3 | YA < MA | hsa-miR-9 | 3 |
| E2F4 | YA > MA | hsa-miR-210 | 4 |
| EFHC1 | YA < MA | hsa-miR-372 | 4 |
|  |  | hsa-miR-33b | 3 |
|  |  | hsa-miR-106a | 4 |
|  |  | hsa-miR-106b | 4 |
|  |  | hsa-miR-18b | 5 |
| EHD4 | YA < MA | hsa-miR-9 | 7 |
| EIF2C4 | YA < MA | hsa-miR-106a | 4 |
|  |  | hsa-miR-106b | 4 |
| EMD | YA > MA | hsa-miR-9 | 5 |
| EPHA3 | YA < MA | hsa-miR-33b | 5 |
| ERBB4 | YA < MA | hsa-miR-9 | 5 |
|  |  | hsa-miR-372 | 3 |
|  |  | hsa-miR-33b | 3 |
|  |  | hsa-miR-106a | 6 |
|  |  | hsa-miR-106b | 5 |
|  |  | hsa-miR-18b | 3 |
| ERCC6 | YA < MA | hsa-miR-33b | 3 |
| ESD | YA > MA | hsa-miR-33b | 3 |
| ESPN | YA < MA | hsa-miR-210 | 4 |
| ESR1 | YA < MA | hsa-miR-9 | 3 |
|  |  | hsa-miR-372 | 4 |
|  |  | hsa-miR-33b | 4 |
|  |  | hsa-miR-33b* | 3 |
|  |  | hsa-miR-106a | 5 |
|  |  | hsa-miR-106b | 5 |
|  |  | hsa-miR-18b | 5 |
|  |  | hsa-miR-18b* | 4 |
|  |  | hsa-miR-18b | 3 |
|  |  | hsa-miR-18b* | 3 |
| FAM101B | YA > MA | hsa-miR-9 | 3 |
|  |  | hsa-miR-33b | 5 |
|  |  | hsa-miR-106a | 5 |
|  |  | hsa-miR-106a* | 3 |
|  |  | hsa-miR-106b | 3 |
| FAM107B | YA < MA | hsa-miR-9 | 7 |
|  |  | hsa-miR-372 | 3 |
|  |  | hsa-miR-33b | 3 |
|  |  | hsa-miR-106a | 4 |
| FAM108C1 | YA < MA | hsa-miR-9 | 4 |
| FAM120AOS | YA < MA | hsa-miR-9 | 5 |
| FAM19A1 | YA < MA | hsa-miR-372 | 4 |
|  |  | hsa-miR-106b | 4 |
| FAM38B | YA > MA | hsa-miR-9 | 3 |
|  |  | hsa-miR-33b | 6 |
|  |  | hsa-miR-33b* | 3 |
| FAM44A | YA < MA | hsa-miR-33b | 3 |
|  |  | hsa-miR-106a | 3 |
|  |  | hsa-miR-106b | 4 |
| FAM53B | YA < MA | hsa-miR-9 | 3 |
|  |  | hsa-miR-372 | 4 |
|  |  | hsa-miR-210 | 4 |
|  |  | hsa-miR-106a | 4 |
|  |  | hsa-miR-106b | 4 |
| FAM76B | YA < MA | hsa-miR-9 | 5 |
|  |  | hsa-miR-33b | 3 |
|  |  | hsa-miR-106a | 4 |
|  |  | hsa-miR-106a* | 3 |
|  |  | hsa-miR-106b | 3 |
|  |  | hsa-miR-18b | 3 |
| FBLN1 | YA > MA | hsa-miR-372 | 4 |
|  |  | hsa-miR-106a | 6 |
|  |  | hsa-miR-106a* | 3 |
|  |  | hsa-miR-106b | 5 |
| FBXL14 | YA < MA | hsa-miR-9 | 5 |
|  |  | hsa-miR-106a | 3 |
|  |  | hsa-miR-106b | 5 |
| FBXO11 | YA > MA | hsa-miR-372 | 6 |
|  |  | hsa-miR-106a | 4 |
|  |  | hsa-miR-106b | 5 |
| FBXO21 | YA < MA | hsa-miR-9 | 3 |
|  |  | hsa-miR-372 | 5 |
|  |  | hsa-miR-33b | 3 |
|  |  | hsa-miR-106a | 6 |
|  |  | hsa-miR-106a* | 3 |
|  |  | hsa-miR-106b | 7 |
|  |  | hsa-miR-18b | 4 |
| FBXO3 | YA < MA | hsa-miR-518a-3p | 6 |
| FBXO33 | YA < MA | hsa-miR-9 | 6 |
|  |  | hsa-miR-33b | 4 |
| FBXO40 | YA < MA | hsa-miR-9 | 4 |
|  |  | hsa-miR-372 | 5 |
|  |  | hsa-miR-33b | 4 |
|  |  | hsa-miR-33b* | 3 |
|  |  | hsa-miR-106a | 6 |
|  |  | hsa-miR-106b | 6 |
|  |  | hsa-miR-18b | 3 |
| FBXO46 | YA < MA | hsa-miR-106a | 3 |
|  |  | hsa-miR-106b | 3 |
| FCAR | YA < MA | hsa-miR-9 | 3 |
| FERMT1 | YA < MA | hsa-miR-9 | 4 |
|  |  | hsa-miR-106a | 5 |
|  |  | hsa-miR-106b | 3 |
|  |  | hsa-miR-18b | 4 |
| FGF1 | YA > MA | hsa-miR-33b | 4 |
|  |  | hsa-miR-33b* | 3 |
|  |  | hsa-miR-210 | 3 |
|  |  | hsa-miR-18b | 3 |
| FKBP6 | YA > MA | hsa-miR-18b | 3 |
| FLJ22167 | YA > MA | hsa-miR-372 | 4 |
|  |  | hsa-miR-106a | 4 |
|  |  | hsa-miR-106b | 3 |
| FOXK2 | YA < MA | hsa-miR-372 | 5 |
|  |  | hsa-miR-106a | 5 |
|  |  | hsa-miR-106b | 5 |
|  |  | hsa-miR-18b | 5 |
| FOXO3 | YA > MA | hsa-miR-9 | 4 |
|  |  | hsa-miR-372 | 5 |
|  |  | hsa-miR-33b | 3 |
|  |  | hsa-miR-18b | 3 |
| FXN | YA < MA | hsa-miR-9 | 5 |
|  |  | hsa-miR-372 | 5 |
|  |  | hsa-miR-33b | 3 |
|  |  | hsa-miR-106a | 5 |
|  |  | hsa-miR-106b | 5 |
| GAD2 | YA < MA | hsa-miR-9 | 3 |
|  |  | hsa-miR-33b | 3 |
|  |  | hsa-miR-106a | 3 |
|  |  | hsa-miR-106b | 3 |
| GALNTL5 | YA < MA | hsa-miR-33b | 3 |
| GALR1 | YA < MA | hsa-miR-9 | 5 |
|  |  | hsa-miR-210 | 4 |
|  |  | hsa-miR-106b | 3 |
| GAPVD1 | YA < MA | hsa-miR-9 | 5 |
|  |  | hsa-miR-33b | 4 |
| GARNL4 | YA < MA | hsa-miR-518a-3p | 4 |
|  |  | hsa-miR-372 | 3 |
|  |  | hsa-miR-106a | 3 |
| GFAP | YA < MA | hsa-miR-372 | 5 |
| GJA9 | YA < MA | hsa-miR-106a | 3 |
|  |  | hsa-miR-106b | 3 |
|  |  | hsa-miR-18b | 3 |
| GLB1L3 | YA < MA | hsa-miR-9 | 3 |
|  |  | hsa-miR-106a | 3 |
|  |  | hsa-miR-106b | 3 |
| GLRX5 | YA > MA | hsa-miR-9 | 5 |
| GLT8D3 | YA > MA | hsa-miR-9 | 4 |
|  |  | hsa-miR-372 | 4 |
|  |  | hsa-miR-33b | 3 |
|  |  | hsa-miR-106a | 4 |
|  |  | hsa-miR-106b | 5 |
|  |  | hsa-miR-18b | 3 |
| GNA12 | YA > MA | hsa-miR-18b | 5 |
| GOLGA8A | YA < MA | hsa-miR-372 | 5 |
|  |  | hsa-miR-33b | 4 |
|  |  | hsa-miR-106a | 3 |
|  |  | hsa-miR-106b | 6 |
| GOSR2 | YA < MA | hsa-miR-106a | 3 |
|  |  | hsa-miR-18b | 4 |
|  |  | hsa-miR-18b* | 3 |
| GPC4 | YA < MA | hsa-miR-9 | 5 |
|  |  | hsa-miR-106a | 4 |
| GRAP | YA < MA | hsa-miR-18b | 5 |
| GREB1 | YA > MA | hsa-miR-9 | 5 |
|  |  | hsa-miR-33b | 3 |
|  |  | hsa-miR-106a | 3 |
|  |  | hsa-miR-106b | 5 |
| GSG1L | YA < MA | hsa-miR-33b | 4 |
|  |  | hsa-miR-33b* | 3 |
| GSTM4 | YA > MA | hsa-miR-106a | 3 |
|  |  | hsa-miR-106b | 3 |
| GTF2E2 | YA > MA | hsa-miR-33b | 3 |
| GTF2H5 | YA < MA | hsa-miR-372 | 4 |
|  |  | hsa-miR-33b | 3 |
|  |  | hsa-miR-33b* | 3 |
|  |  | hsa-miR-106a | 3 |
|  |  | hsa-miR-106a* | 3 |
|  |  | hsa-miR-106b | 3 |
| GTPBP2 | YA > MA | hsa-miR-9 | 7 |
|  |  | hsa-miR-372 | 3 |
| GTSF1L | YA < MA | hsa-miR-33b | 5 |
| GUCA1B | YA < MA | hsa-miR-372 | 6 |
|  |  | hsa-miR-106a | 6 |
|  |  | hsa-miR-106b | 6 |
| GUCY1A2 | YA > MA | hsa-miR-106a | 4 |
| GYPA | YA < MA | hsa-miR-33b | 3 |
| HAPLN4 | YA < MA | hsa-miR-106a | 3 |
|  |  | hsa-miR-18b | 4 |
| HCFC2 | YA < MA | hsa-miR-18b | 3 |
| HDDC2 | YA > MA | hsa-miR-33b | 3 |
| HDGF | YA > MA | hsa-miR-106a | 4 |
| HDLBP | YA > MA | hsa-miR-210 | 3 |
| HEPH | YA > MA | hsa-miR-106a | 3 |
| HERC3 | YA < MA | hsa-miR-18b | 5 |
| HERV-FRD | YA < MA | hsa-miR-9 | 4 |
|  |  | hsa-miR-372 | 3 |
|  |  | hsa-miR-106a | 4 |
|  |  | hsa-miR-106b | 4 |
| HHLA2 | YA < MA | hsa-miR-9 | 4 |
|  |  | hsa-miR-18b | 4 |
| HIATL1 | YA < MA | hsa-miR-210 | 5 |
| HIBCH | YA < MA | hsa-miR-106b | 3 |
| HIF1AN | YA > MA | hsa-miR-372 | 5 |
|  |  | hsa-miR-33b | 3 |
|  |  | hsa-miR-106a | 4 |
|  |  | hsa-miR-106b | 4 |
|  |  | hsa-miR-18b | 5 |
|  |  | hsa-miR-18b* | 3 |
| HIGD1A | YA > MA | hsa-miR-9 | 3 |
|  |  | hsa-miR-33b | 5 |
|  |  | hsa-miR-106a | 3 |
|  |  | hsa-miR-106b | 3 |
| HNF4G | YA < MA | hsa-miR-9 | 5 |
|  |  | hsa-miR-372 | 6 |
|  |  | hsa-miR-106a | 6 |
|  |  | hsa-miR-106b | 5 |
| HNRNPA2B1 | YA > MA | hsa-miR-33b | 5 |
| HNRNPL | YA > MA | hsa-miR-106a | 4 |
| HNRPDL | YA > MA | hsa-miR-18b | 3 |
| HPCAL4 | YA < MA | hsa-miR-9 | 3 |
|  |  | hsa-miR-372 | 3 |
|  |  | hsa-miR-106a | 5 |
|  |  | hsa-miR-106b | 5 |
| HS6ST2 | YA > MA | hsa-miR-518a-3p | 5 |
|  |  | hsa-miR-18b | 5 |
| HSDL2 | YA < MA | hsa-miR-18b | 5 |
| HSP90AA1 | YA > MA | hsa-miR-9 | 6 |
| HTR2C | YA < MA | hsa-miR-106a | 5 |
|  |  | hsa-miR-106a* | 3 |
|  |  | hsa-miR-106b | 6 |
| HTRA2 | YA > MA | hsa-miR-106a | 4 |
| ICA1L | YA < MA | hsa-miR-9 | 5 |
|  |  | hsa-miR-372 | 5 |
|  |  | hsa-miR-33b | 5 |
|  |  | hsa-miR-106a | 5 |
|  |  | hsa-miR-106a* | 3 |
|  |  | hsa-miR-106b | 5 |
|  |  | hsa-miR-18b | 5 |
| IKZF4 | YA < MA | hsa-miR-9 | 6 |
|  |  | hsa-miR-372 | 5 |
|  |  | hsa-miR-106a | 5 |
|  |  | hsa-miR-106b | 7 |
| IL16 | YA < MA | hsa-miR-9 | 4 |
|  |  | hsa-miR-372 | 4 |
|  |  | hsa-miR-33b | 3 |
|  |  | hsa-miR-210 | 4 |
|  |  | hsa-miR-106a | 4 |
|  |  | hsa-miR-106a* | 3 |
| IL28RA | YA < MA | hsa-miR-372 | 6 |
|  |  | hsa-miR-106a | 3 |
|  |  | hsa-miR-106b | 3 |
|  |  | hsa-miR-18b | 4 |
|  |  | hsa-miR-18b* | 3 |
| IL29 | YA > MA | hsa-miR-18b | 3 |
| ILK | YA > MA | hsa-miR-33b | 3 |
| IQCC | YA < MA | hsa-miR-372 | 5 |
|  |  | hsa-miR-106a | 5 |
|  |  | hsa-miR-106b | 5 |
| IQCK | YA < MA | hsa-miR-33b | 3 |
|  |  | hsa-miR-106a | 3 |
| IRAK4 | YA < MA | hsa-miR-372 | 4 |
|  |  | hsa-miR-106b | 4 |
| ISL2 | YA < MA | hsa-miR-9 | 3 |
| ITGAD | YA > MA | hsa-miR-106a | 4 |
|  |  | hsa-miR-18b | 5 |
| ITPR1 | YA < MA | hsa-miR-106a | 3 |
|  |  | hsa-miR-106b | 3 |
| JMJD2C | YA < MA | hsa-miR-9 | 5 |
|  |  | hsa-miR-106a | 3 |
|  |  | hsa-miR-106a* | 3 |
| KCND1 | YA < MA | hsa-miR-33b | 3 |
| KCNJ10 | YA < MA | hsa-miR-372 | 5 |
|  |  | hsa-miR-33b | 3 |
|  |  | hsa-miR-106a | 5 |
|  |  | hsa-miR-106b | 6 |
| KCNJ13 | YA < MA | hsa-miR-106a | 3 |
| KCNK5 | YA < MA | hsa-miR-33b | 5 |
|  |  | hsa-miR-210 | 5 |
|  |  | hsa-miR-18b | 3 |
| KIAA0174 | YA > MA | hsa-miR-9 | 5 |
|  |  | hsa-miR-33b | 3 |
| KIAA0494 | YA < MA | hsa-miR-372 | 5 |
|  |  | hsa-miR-106a | 6 |
|  |  | hsa-miR-106b | 6 |
|  |  | hsa-miR-18b | 5 |
| KIAA1045 | YA < MA | hsa-miR-9 | 6 |
|  |  | hsa-miR-518a-3p | 4 |
|  |  | hsa-miR-18b | 6 |
| KIAA1147 | YA < MA | hsa-miR-9 | 3 |
|  |  | hsa-miR-372 | 5 |
|  |  | hsa-miR-33b | 5 |
|  |  | hsa-miR-106a | 6 |
|  |  | hsa-miR-106b | 6 |
|  |  | hsa-miR-18b | 4 |
| KIAA1199 | YA < MA | hsa-miR-9 | 5 |
|  |  | hsa-miR-33b | 3 |
|  |  | hsa-miR-18b | 6 |
|  |  | hsa-miR-18b* | 3 |
| KIAA1529 | YA < MA | hsa-miR-106a | 3 |
|  |  | hsa-miR-106b | 3 |
| KIF20A | YA > MA | hsa-miR-106a | 4 |
| KIF6 | YA < MA | hsa-miR-33b | 5 |
| KLF6 | YA < MA | hsa-miR-372 | 3 |
|  |  | hsa-miR-18b | 5 |
| KLHL21 | YA < MA | hsa-miR-372 | 4 |
|  |  | hsa-miR-106a | 3 |
|  |  | hsa-miR-106b | 3 |
| KLHL23 | YA < MA | hsa-miR-106a | 4 |
| KLHL4 | YA < MA | hsa-miR-9 | 4 |
|  |  | hsa-miR-33b | 5 |
|  |  | hsa-miR-18b | 4 |
| KLHL9 | YA < MA | hsa-miR-9 | 5 |
| KLK2 | YA < MA | hsa-miR-9 | 5 |
|  |  | hsa-miR-33b | 5 |
|  |  | hsa-miR-33b* | 3 |
| KRTAP9-9 | YA < MA | hsa-miR-106a | 3 |
|  |  | hsa-miR-106b | 3 |
| KY | YA < MA | hsa-miR-372 | 4 |
|  |  | hsa-miR-33b | 3 |
|  |  | hsa-miR-210 | 4 |
|  |  | hsa-miR-106a | 3 |
|  |  | hsa-miR-106b | 4 |
| L1TD1 | YA > MA | hsa-miR-372 | 4 |
|  |  | hsa-miR-106a | 4 |
|  |  | hsa-miR-106b | 4 |
| L3MBTL | YA < MA | hsa-miR-106b | 4 |
| LACE1 | YA < MA | hsa-miR-372 | 6 |
|  |  | hsa-miR-33b | 5 |
|  |  | hsa-miR-106a | 6 |
|  |  | hsa-miR-106b | 8 |
|  |  | hsa-miR-106b* | 3 |
| LAGE3 | YA > MA | hsa-miR-372 | 3 |
| LAMC2 | YA > MA | hsa-miR-9 | 7 |
| LAMC3 | YA > MA | hsa-miR-9 | 5 |
|  |  | hsa-miR-518a-3p | 4 |
|  |  | hsa-miR-33b | 3 |
| LBA1 | YA < MA | hsa-miR-9 | 3 |
| LDB3 | YA < MA | hsa-miR-9 | 5 |
|  |  | hsa-miR-372 | 5 |
|  |  | hsa-miR-106a | 5 |
|  |  | hsa-miR-106a* | 3 |
|  |  | hsa-miR-106b | 5 |
| LDHAL6B | YA < MA | hsa-miR-372 | 5 |
| LHPP | YA < MA | hsa-miR-18b | 3 |
| LIF | YA < MA | hsa-miR-9 | 4 |
|  |  | hsa-miR-372 | 5 |
|  |  | hsa-miR-106a | 6 |
|  |  | hsa-miR-106b | 6 |
|  |  | hsa-miR-18b | 6 |
|  |  | hsa-miR-18b* | 3 |
| LIPH | YA < MA | hsa-miR-106a | 3 |
|  |  | hsa-miR-106b | 5 |
| LONP2 | YA < MA | hsa-miR-372 | 6 |
|  |  | hsa-miR-106a | 7 |
|  |  | hsa-miR-106a* | 3 |
|  |  | hsa-miR-106b | 7 |
| LRP11 | YA > MA | hsa-miR-518a-3p | 3 |
| LRRC45 | YA > MA | hsa-miR-372 | 4 |
|  |  | hsa-miR-106a | 6 |
|  |  | hsa-miR-106b | 6 |
| LRRC57 | YA < MA | hsa-miR-372 | 4 |
|  |  | hsa-miR-106a | 5 |
|  |  | hsa-miR-106b | 5 |
| LTV1 | YA < MA | hsa-miR-372 | 4 |
|  |  | hsa-miR-106a | 5 |
|  |  | hsa-miR-106b | 5 |
| MAF1 | YA > MA | hsa-miR-106a | 3 |
| MAGI2 | YA < MA | hsa-miR-372 | 4 |
|  |  | hsa-miR-33b | 3 |
|  |  | hsa-miR-210 | 3 |
|  |  | hsa-miR-106a | 5 |
|  |  | hsa-miR-106a* | 3 |
|  |  | hsa-miR-106b | 5 |
|  |  | hsa-miR-18b | 3 |
| MANEA | YA < MA | hsa-miR-372 | 6 |
|  |  | hsa-miR-33b | 6 |
|  |  | hsa-miR-106a | 3 |
|  |  | hsa-miR-106b | 3 |
| MAP3K7IP2 | YA < MA | hsa-miR-9 | 3 |
| MAPT | YA < MA | hsa-miR-372 | 4 |
| MCAM | YA > MA | hsa-miR-372 | 4 |
|  |  | hsa-miR-106a | 4 |
|  |  | hsa-miR-106b | 4 |
| MDGA1 | YA < MA | hsa-miR-9 | 7 |
|  |  | hsa-miR-33b | 3 |
|  |  | hsa-miR-210 | 6 |
|  |  | hsa-miR-18b | 6 |
|  |  | hsa-miR-18b* | 4 |
| MECR | YA < MA | hsa-miR-372 | 4 |
|  |  | hsa-miR-106a | 4 |
|  |  | hsa-miR-106b | 4 |
| MED8 | YA < MA | hsa-miR-9 | 5 |
|  |  | hsa-miR-18b | 5 |
| METRNL | YA < MA | hsa-miR-33b | 3 |
| METTL8 | YA < MA | hsa-miR-372 | 5 |
|  |  | hsa-miR-33b | 5 |
|  |  | hsa-miR-33b* | 3 |
|  |  | hsa-miR-106a | 5 |
|  |  | hsa-miR-106a* | 3 |
|  |  | hsa-miR-106b | 5 |
|  |  | hsa-miR-18b | 5 |
| MFSD11 | YA < MA | hsa-miR-106a | 3 |
|  |  | hsa-miR-106b | 3 |
| MGA | YA > MA | hsa-miR-9 | 3 |
|  |  | hsa-miR-372 | 4 |
|  |  | hsa-miR-106a | 4 |
| MGAT1 | YA > MA | hsa-miR-9 | 4 |
| MICB | YA > MA | hsa-miR-372 | 4 |
|  |  | hsa-miR-106b | 4 |
| MKNK2 | YA < MA | hsa-miR-9 | 5 |
|  |  | hsa-miR-372 | 4 |
|  |  | hsa-miR-106a | 6 |
|  |  | hsa-miR-106a* | 3 |
|  |  | hsa-miR-106b | 6 |
|  |  | hsa-miR-106b* | 3 |
| MLC1 | YA < MA | hsa-miR-372 | 3 |
|  |  | hsa-miR-210 | 4 |
|  |  | hsa-miR-106a | 4 |
|  |  | hsa-miR-106b | 4 |
| MLL5 | YA > MA | hsa-miR-106a | 3 |
|  |  | hsa-miR-106b* | 4 |
|  |  | hsa-miR-106b | 3 |
|  |  | hsa-miR-106b* | 3 |
|  |  | hsa-miR-18b | 3 |
| MOBKL1A | YA < MA | hsa-miR-372 | 3 |
|  |  | hsa-miR-106a | 6 |
|  |  | hsa-miR-106a* | 3 |
|  |  | hsa-miR-106b | 5 |
|  |  | hsa-miR-18b | 5 |
| MOGAT3 | YA > MA | hsa-miR-372 | 6 |
|  |  | hsa-miR-106a | 6 |
|  |  | hsa-miR-106b | 6 |
| MRFAP1 | YA > MA | hsa-miR-9 | 5 |
| MRPL30 | YA < MA | hsa-miR-9 | 3 |
|  |  | hsa-miR-372 | 5 |
|  |  | hsa-miR-33b | 3 |
|  |  | hsa-miR-106a | 5 |
|  |  | hsa-miR-106b | 5 |
|  |  | hsa-miR-18b | 5 |
| MRPS10 | YA < MA | hsa-miR-372 | 3 |
|  |  | hsa-miR-210 | 3 |
|  |  | hsa-miR-106a | 4 |
|  |  | hsa-miR-106b | 3 |
| MTCP1 | YA > MA | hsa-miR-372 | 6 |
|  |  | hsa-miR-18b | 5 |
| MTM1 | YA > MA | hsa-miR-9 | 5 |
|  |  | hsa-miR-33b | 4 |
| MTMR15 | YA < MA | hsa-miR-106a | 4 |
| MUC17 | YA < MA | hsa-miR-372 | 6 |
|  |  | hsa-miR-106a | 6 |
|  |  | hsa-miR-106b | 6 |
|  |  | hsa-miR-18b | 3 |
| MUT | YA < MA | hsa-miR-9 | 3 |
| MXRA7 | YA > MA | hsa-miR-210 | 3 |
| MXRA8 | YA > MA | hsa-miR-518a-3p | 4 |
| MYCL1 | YA > MA | hsa-miR-106b | 4 |
| MYCT1 | YA > MA | hsa-miR-9 | 3 |
|  |  | hsa-miR-372 | 5 |
|  |  | hsa-miR-106a | 5 |
|  |  | hsa-miR-106a* | 3 |
|  |  | hsa-miR-106b | 5 |
| MYF5 | YA < MA | hsa-miR-106a | 3 |
| MYH4 | YA < MA | hsa-miR-9 | 5 |
|  |  | hsa-miR-33b | 4 |
| MYL9 | YA > MA | hsa-miR-33b | 3 |
|  |  | hsa-miR-210 | 3 |
| MYO16 | YA < MA | hsa-miR-33b | 3 |
| MYO1B | YA < MA | hsa-miR-9 | 4 |
| MYT1L | YA < MA | hsa-miR-372 | 6 |
|  |  | hsa-miR-33b | 3 |
|  |  | hsa-miR-210 | 5 |
|  |  | hsa-miR-106a | 6 |
|  |  | hsa-miR-106b | 6 |
| NAPEPLD | YA < MA | hsa-miR-372 | 5 |
|  |  | hsa-miR-33b | 4 |
|  |  | hsa-miR-106a | 5 |
|  |  | hsa-miR-106b | 5 |
|  |  | hsa-miR-18b | 4 |
| NCAM1 | YA < MA | hsa-miR-372 | 3 |
|  |  | hsa-miR-33b | 5 |
|  |  | hsa-miR-33b* | 3 |
|  |  | hsa-miR-210 | 5 |
|  |  | hsa-miR-106a | 4 |
|  |  | hsa-miR-106a* | 3 |
|  |  | hsa-miR-18b | 3 |
| NCBP2 | YA < MA | hsa-miR-106a | 4 |
| NCOA5 | YA > MA | hsa-miR-372 | 4 |
|  |  | hsa-miR-106a | 3 |
|  |  | hsa-miR-106b | 3 |
|  |  | hsa-miR-18b | 3 |
| NDUFA10 | YA > MA | hsa-miR-33b | 3 |
| NETO1 | YA > MA | hsa-miR-106a | 3 |
| NFE2L1 | YA < MA | hsa-miR-9 | 3 |
|  |  | hsa-miR-518a-3p | 5 |
|  |  | hsa-miR-210 | 4 |
|  |  | hsa-miR-106a | 3 |
|  |  | hsa-miR-106b | 4 |
| NICN1 | YA < MA | hsa-miR-372 | 5 |
|  |  | hsa-miR-210 | 4 |
|  |  | hsa-miR-106a | 5 |
|  |  | hsa-miR-106a* | 3 |
|  |  | hsa-miR-106b | 5 |
| NIP30 | YA > MA | hsa-miR-9 | 3 |
|  |  | hsa-miR-18b | 5 |
| NKTR | YA < MA | hsa-miR-9 | 5 |
| NOD2 | YA < MA | hsa-miR-18b | 5 |
| NOS1AP | YA > MA | hsa-miR-372 | 4 |
|  |  | hsa-miR-106a | 4 |
|  |  | hsa-miR-106a* | 3 |
|  |  | hsa-miR-106b | 4 |
| NPC1L1 | YA > MA | hsa-miR-9 | 5 |
|  |  | hsa-miR-18b | 5 |
| NRSN2 | YA > MA | hsa-miR-18b | 3 |
| NSFL1C | YA > MA | hsa-miR-9 | 3 |
| NSUN7 | YA < MA | hsa-miR-9 | 5 |
|  |  | hsa-miR-33b | 3 |
| NT5DC2 | YA > MA | hsa-miR-18b | 3 |
| NUFIP1 | YA > MA | hsa-miR-33b | 3 |
| NUP210 | YA > MA | hsa-miR-210 | 3 |
|  |  | hsa-miR-106a | 3 |
|  |  | hsa-miR-106b | 3 |
| NXPH3 | YA > MA | hsa-miR-18b | 3 |
| OGFRL1 | YA < MA | hsa-miR-106a | 3 |
| OLAH | YA < MA | hsa-miR-372 | 4 |
|  |  | hsa-miR-106a | 4 |
|  |  | hsa-miR-106b | 4 |
| OPRM1 | YA < MA | hsa-miR-9 | 3 |
|  |  | hsa-miR-18b | 5 |
| OR2H1 | YA < MA | hsa-miR-106a | 4 |
| ORC6L | YA > MA | hsa-miR-372 | 6 |
|  |  | hsa-miR-106a | 6 |
|  |  | hsa-miR-106b | 6 |
| OSBPL10 | YA < MA | hsa-miR-106a | 3 |
| PANX3 | YA < MA | hsa-miR-210 | 5 |
| PAPOLA | YA > MA | hsa-miR-372 | 7 |
|  |  | hsa-miR-106a | 7 |
|  |  | hsa-miR-106b | 8 |
|  |  | hsa-miR-106b* | 3 |
| PAQR3 | YA < MA | hsa-miR-372 | 3 |
|  |  | hsa-miR-33b | 3 |
| PARP12 | YA < MA | hsa-miR-106a | 4 |
| PBK | YA > MA | hsa-miR-372 | 6 |
|  |  | hsa-miR-106a | 7 |
|  |  | hsa-miR-106b | 7 |
| PBLD | YA < MA | hsa-miR-372 | 5 |
|  |  | hsa-miR-106a | 7 |
|  |  | hsa-miR-106b | 6 |
| PCM1 | YA > MA | hsa-miR-9 | 3 |
|  |  | hsa-miR-33b | 6 |
|  |  | hsa-miR-106a | 3 |
|  |  | hsa-miR-106b | 4 |
|  |  | hsa-miR-18b | 4 |
| PCMTD1 | YA < MA | hsa-miR-518a-3p | 3 |
|  |  | hsa-miR-372 | 4 |
|  |  | hsa-miR-33b | 3 |
|  |  | hsa-miR-106a | 5 |
|  |  | hsa-miR-106a* | 3 |
|  |  | hsa-miR-106b | 5 |
| PCSK6 | YA < MA | hsa-miR-9 | 5 |
|  |  | hsa-miR-33b | 4 |
|  |  | hsa-miR-33b* | 3 |
| PCYT1B | YA < MA | hsa-miR-372 | 5 |
|  |  | hsa-miR-210 | 5 |
|  |  | hsa-miR-106a | 5 |
|  |  | hsa-miR-106b | 5 |
| PECR | YA < MA | hsa-miR-372 | 6 |
|  |  | hsa-miR-106a | 6 |
|  |  | hsa-miR-106b | 6 |
| PER2 | YA > MA | hsa-miR-9 | 4 |
|  |  | hsa-miR-372 | 5 |
|  |  | hsa-miR-33b | 4 |
|  |  | hsa-miR-106a | 5 |
|  |  | hsa-miR-106b | 5 |
|  |  | hsa-miR-18b | 3 |
| PEX19 | YA < MA | hsa-miR-372 | 4 |
|  |  | hsa-miR-106a | 4 |
|  |  | hsa-miR-106b | 4 |
| PHB | YA > MA | hsa-miR-9 | 5 |
| PHF14 | YA > MA | hsa-miR-33b | 3 |
| PHF15 | YA < MA | hsa-miR-9 | 5 |
|  |  | hsa-miR-372 | 5 |
|  |  | hsa-miR-210 | 4 |
|  |  | hsa-miR-106a | 3 |
|  |  | hsa-miR-106b | 3 |
|  |  | hsa-miR-18b | 3 |
| PHF20 | YA > MA | hsa-miR-9 | 3 |
|  |  | hsa-miR-372 | 5 |
|  |  | hsa-miR-33b | 4 |
|  |  | hsa-miR-106a | 5 |
|  |  | hsa-miR-106a* | 3 |
|  |  | hsa-miR-106b | 5 |
| PHF20L1 | YA < MA | hsa-miR-9 | 6 |
|  |  | hsa-miR-372 | 5 |
|  |  | hsa-miR-33b | 3 |
|  |  | hsa-miR-18b | 5 |
|  |  | hsa-miR-18b* | 3 |
| PIGG | YA < MA | hsa-miR-106b | 3 |
| PIGK | YA < MA | hsa-miR-9 | 5 |
|  |  | hsa-miR-372 | 3 |
|  |  | hsa-miR-33b | 3 |
|  |  | hsa-miR-106a | 4 |
|  |  | hsa-miR-106b | 5 |
| PIGL | YA < MA | hsa-miR-9 | 4 |
| PIGS | YA > MA | hsa-miR-9 | 5 |
|  |  | hsa-miR-372 | 3 |
| PIP3-E | YA < MA | hsa-miR-9 | 4 |
|  |  | hsa-miR-106a | 4 |
|  |  | hsa-miR-106a* | 3 |
|  |  | hsa-miR-106b | 4 |
| PIP5K1C | YA < MA | hsa-miR-18b | 6 |
| PKP1 | YA > MA | hsa-miR-372 | 4 |
|  |  | hsa-miR-33b | 4 |
| PLA2G2D | YA > MA | hsa-miR-9 | 5 |
| PLEKHA1 | YA < MA | hsa-miR-9 | 6 |
|  |  | hsa-miR-106a | 5 |
|  |  | hsa-miR-106a* | 3 |
|  |  | hsa-miR-106b | 5 |
| PLEKHA3 | YA < MA | hsa-miR-372 | 6 |
|  |  | hsa-miR-106a | 6 |
|  |  | hsa-miR-106b | 6 |
| PLEKHA7 | YA < MA | hsa-miR-9 | 5 |
|  |  | hsa-miR-18b | 5 |
| PLLP | YA > MA | hsa-miR-33b | 4 |
| PODN | YA > MA | hsa-miR-372 | 4 |
|  |  | hsa-miR-106a | 5 |
|  |  | hsa-miR-106b | 5 |
| POF1B | YA < MA | hsa-miR-33b | 5 |
|  |  | hsa-miR-33b* | 3 |
|  |  | hsa-miR-106a | 3 |
|  |  | hsa-miR-106b | 4 |
| POLH | YA < MA | hsa-miR-372 | 5 |
|  |  | hsa-miR-33b | 4 |
|  |  | hsa-miR-106a | 5 |
|  |  | hsa-miR-106a* | 3 |
|  |  | hsa-miR-106b | 5 |
| POLI | YA < MA | hsa-miR-9 | 5 |
| POLQ | YA > MA | hsa-miR-372 | 6 |
|  |  | hsa-miR-106a | 7 |
|  |  | hsa-miR-106b | 7 |
| PPAP2A | YA > MA | hsa-miR-106a | 3 |
| PPM1A | YA > MA | hsa-miR-106a | 3 |
| PPM1L | YA < MA | hsa-miR-9 | 3 |
|  |  | hsa-miR-372 | 5 |
| PPM1M | YA > MA | hsa-miR-9 | 4 |
| PPP1R1A | YA > MA | hsa-miR-372 | 4 |
|  |  | hsa-miR-106a | 5 |
|  |  | hsa-miR-106b | 5 |
| PPP1R3A | YA < MA | hsa-miR-33b | 4 |
| PPP2R3A | YA < MA | hsa-miR-372 | 5 |
|  |  | hsa-miR-33b | 3 |
|  |  | hsa-miR-106a | 5 |
|  |  | hsa-miR-106b | 6 |
| PPP2R5C | YA > MA | hsa-miR-106a | 3 |
| PRIM2 | YA < MA | hsa-miR-18b | 3 |
| PRIMA1 | YA < MA | hsa-miR-33b | 5 |
|  |  | hsa-miR-33b* | 3 |
|  |  | hsa-miR-18b | 5 |
| PRKACA | YA > MA | hsa-miR-9 | 6 |
|  |  | hsa-miR-106a | 4 |
| PRKAG2 | YA < MA | hsa-miR-106a | 4 |
| PRKAR1B | YA < MA | hsa-miR-106a | 3 |
|  |  | hsa-miR-18b | 3 |
| PRRC1 | YA > MA | hsa-miR-9 | 5 |
|  |  | hsa-miR-106a | 5 |
|  |  | hsa-miR-106b | 5 |
| PRSS23 | YA > MA | hsa-miR-372 | 5 |
|  |  | hsa-miR-33b | 3 |
|  |  | hsa-miR-18b | 6 |
| PTBP2 | YA < MA | hsa-miR-9 | 7 |
| PTPDC1 | YA < MA | hsa-miR-9 | 3 |
|  |  | hsa-miR-372 | 4 |
|  |  | hsa-miR-106a | 4 |
|  |  | hsa-miR-106b | 5 |
| PTPN2 | YA < MA | hsa-miR-372 | 3 |
|  |  | hsa-miR-106a | 4 |
| PTPN9 | YA > MA | hsa-miR-372 | 3 |
|  |  | hsa-miR-33b | 3 |
| PTPRF | YA < MA | hsa-miR-106a | 3 |
|  |  | hsa-miR-106b | 3 |
| PVRL1 | YA > MA | hsa-miR-9 | 3 |
|  |  | hsa-miR-372 | 5 |
|  |  | hsa-miR-18b | 5 |
| PWWP2A | YA < MA | hsa-miR-372 | 3 |
| PYDC1 | YA < MA | hsa-miR-33b | 3 |
| RABEPK | YA < MA | hsa-miR-33b | 3 |
| RABGAP1 | YA < MA | hsa-miR-372 | 7 |
|  |  | hsa-miR-106a | 5 |
|  |  | hsa-miR-106b | 6 |
|  |  | hsa-miR-18b | 7 |
| RACGAP1 | YA > MA | hsa-miR-372 | 4 |
|  |  | hsa-miR-106a | 5 |
|  |  | hsa-miR-106b | 4 |
| RALGPS1 | YA < MA | hsa-miR-106a | 3 |
| RANBP9 | YA > MA | hsa-miR-518a-3p | 3 |
| RAPGEF6 | YA < MA | hsa-miR-9 | 4 |
|  |  | hsa-miR-33b | 3 |
|  |  | hsa-miR-106b | 3 |
| RBM15B | YA > MA | hsa-miR-106a | 3 |
|  |  | hsa-miR-106b* | 4 |
| RBM4 | YA > MA | hsa-miR-9 | 3 |
|  |  | hsa-miR-106a | 4 |
| RBM43 | YA < MA | hsa-miR-372 | 5 |
|  |  | hsa-miR-106b | 6 |
| RBM46 | YA < MA | hsa-miR-9 | 4 |
|  |  | hsa-miR-372 | 6 |
|  |  | hsa-miR-33b | 3 |
| RCE1 | YA > MA | hsa-miR-9 | 4 |
| RELB | YA > MA | hsa-miR-18b | 5 |
| RERG | YA < MA | hsa-miR-33b | 5 |
|  |  | hsa-miR-18b | 5 |
| RET | YA < MA | hsa-miR-9 | 3 |
|  |  | hsa-miR-106a | 3 |
|  |  | hsa-miR-106b | 5 |
| RFC2 | YA > MA | hsa-miR-106b | 3 |
| RFNG | YA > MA | hsa-miR-372 | 5 |
| RFT1 | YA < MA | hsa-miR-9 | 3 |
|  |  | hsa-miR-372 | 3 |
|  |  | hsa-miR-106a | 4 |
|  |  | hsa-miR-106b | 4 |
|  |  | hsa-miR-18b | 5 |
| RFWD3 | YA > MA | hsa-miR-9 | 5 |
| RHBDL2 | YA < MA | hsa-miR-372 | 5 |
|  |  | hsa-miR-106a | 5 |
|  |  | hsa-miR-106b | 5 |
| RHOC | YA > MA | hsa-miR-372 | 5 |
|  |  | hsa-miR-106a | 5 |
|  |  | hsa-miR-106b | 6 |
| RNF141 | YA < MA | hsa-miR-372 | 6 |
|  |  | hsa-miR-33b | 5 |
|  |  | hsa-miR-33b* | 3 |
|  |  | hsa-miR-106a | 6 |
|  |  | hsa-miR-106b | 6 |
|  |  | hsa-miR-18b | 3 |
| RNF149 | YA < MA | hsa-miR-372 | 4 |
|  |  | hsa-miR-33b | 3 |
|  |  | hsa-miR-106a | 5 |
|  |  | hsa-miR-106b | 5 |
| RNF41 | YA > MA | hsa-miR-18b | 4 |
| ROR2 | YA < MA | hsa-miR-9 | 5 |
|  |  | hsa-miR-106a | 4 |
|  |  | hsa-miR-18b | 3 |
| RPL10 | YA > MA | hsa-miR-9 | 5 |
| RPRM | YA < MA | hsa-miR-33b | 3 |
| RPS6KA1 | YA < MA | hsa-miR-372 | 5 |
|  |  | hsa-miR-106a | 4 |
|  |  | hsa-miR-106b | 5 |
| RRP1B | YA > MA | hsa-miR-9 | 3 |
|  |  | hsa-miR-372 | 3 |
|  |  | hsa-miR-33b | 4 |
|  |  | hsa-miR-33b* | 3 |
|  |  | hsa-miR-210 | 5 |
| RTP4 | YA < MA | hsa-miR-106a | 3 |
|  |  | hsa-miR-106b | 5 |
| RUFY2 | YA < MA | hsa-miR-372 | 4 |
|  |  | hsa-miR-33b | 4 |
|  |  | hsa-miR-106a | 6 |
|  |  | hsa-miR-106b | 6 |
| RXRG | YA < MA | hsa-miR-372 | 4 |
|  |  | hsa-miR-106a | 4 |
|  |  | hsa-miR-106b | 4 |
| RYR2 | YA < MA | hsa-miR-372 | 4 |
| SCRN1 | YA < MA | hsa-miR-9 | 3 |
|  |  | hsa-miR-18b | 4 |
| SCRN3 | YA < MA | hsa-miR-372 | 5 |
|  |  | hsa-miR-33b | 4 |
|  |  | hsa-miR-106a | 6 |
|  |  | hsa-miR-106b | 5 |
| SCYL2 | YA < MA | hsa-miR-106a | 4 |
|  |  | hsa-miR-106a* | 3 |
| SDHC | YA > MA | hsa-miR-106a | 3 |
|  |  | hsa-miR-106b | 5 |
| SEC61A1 | YA > MA | hsa-miR-18b | 6 |
| SERPINA10 | YA < MA | hsa-miR-372 | 4 |
|  |  | hsa-miR-33b | 5 |
| SETD5 | YA < MA | hsa-miR-372 | 3 |
|  |  | hsa-miR-106a | 4 |
|  |  | hsa-miR-106b | 3 |
| SETD6 | YA > MA | hsa-miR-9 | 4 |
| SF1 | YA < MA | hsa-miR-9 | 5 |
| SF3B1 | YA > MA | hsa-miR-33b | 4 |
|  |  | hsa-miR-18b | 5 |
| SFRS2B | YA < MA | hsa-miR-9 | 4 |
|  |  | hsa-miR-372 | 4 |
|  |  | hsa-miR-33b | 4 |
|  |  | hsa-miR-106a | 4 |
|  |  | hsa-miR-106b | 4 |
| SGSM1 | YA < MA | hsa-miR-9 | 6 |
|  |  | hsa-miR-372 | 6 |
|  |  | hsa-miR-33b | 4 |
|  |  | hsa-miR-106a | 6 |
|  |  | hsa-miR-106b | 6 |
|  |  | hsa-miR-106b* | 3 |
| SH3BP5 | YA > MA | hsa-miR-372 | 6 |
|  |  | hsa-miR-106a | 5 |
|  |  | hsa-miR-106b | 6 |
| SHC4 | YA > MA | hsa-miR-372 | 6 |
|  |  | hsa-miR-106a | 4 |
|  |  | hsa-miR-106b | 4 |
| SIRPB1 | YA < MA | hsa-miR-18b | 5 |
| SLC10A7 | YA < MA | hsa-miR-372 | 5 |
|  |  | hsa-miR-33b | 6 |
|  |  | hsa-miR-106a | 5 |
|  |  | hsa-miR-106b | 5 |
|  |  | hsa-miR-18b | 5 |
| SLC25A12 | YA < MA | hsa-miR-372 | 3 |
|  |  | hsa-miR-33b | 4 |
| SLC25A35 | YA < MA | hsa-miR-18b | 3 |
| SLC25A39 | YA > MA | hsa-miR-372 | 3 |
| SLC26A8 | YA < MA | hsa-miR-9 | 5 |
| SLC27A4 | YA < MA | hsa-miR-9 | 6 |
| SLC2A5 | YA < MA | hsa-miR-33b | 3 |
|  |  | hsa-miR-106a | 3 |
| SLC38A1 | YA < MA | hsa-miR-372 | 4 |
|  |  | hsa-miR-210 | 4 |
|  |  | hsa-miR-106a | 4 |
|  |  | hsa-miR-106b | 4 |
| SLC46A2 | YA < MA | hsa-miR-372 | 5 |
|  |  | hsa-miR-106a | 5 |
|  |  | hsa-miR-106b | 5 |
| SLC4A1 | YA < MA | hsa-miR-9 | 7 |
|  |  | hsa-miR-372 | 3 |
|  |  | hsa-miR-106a | 4 |
|  |  | hsa-miR-106b | 4 |
| SLC6A15 | YA < MA | hsa-miR-372 | 3 |
|  |  | hsa-miR-33b | 4 |
|  |  | hsa-miR-33b* | 3 |
| SLC6A16 | YA > MA | hsa-miR-106a | 4 |
| SLC6A3 | YA < MA | hsa-miR-33b | 3 |
|  |  | hsa-miR-18b | 3 |
| SLC6A8 | YA > MA | hsa-miR-106a | 4 |
| SLC8A3 | YA < MA | hsa-miR-9 | 3 |
|  |  | hsa-miR-210 | 5 |
| SMAD3 | YA < MA | hsa-miR-9 | 3 |
|  |  | hsa-miR-372 | 4 |
|  |  | hsa-miR-106a | 3 |
|  |  | hsa-miR-18b | 4 |
| SNX13 | YA > MA | hsa-miR-106a* | 3 |
|  |  | hsa-miR-106b | 3 |
|  |  | hsa-miR-18b | 4 |
| SNX3 | YA > MA | hsa-miR-9 | 3 |
| SOAT1 | YA < MA | hsa-miR-9 | 5 |
| SORBS1 | YA < MA | hsa-miR-33b | 3 |
|  |  | hsa-miR-210 | 5 |
|  |  | hsa-miR-106a | 4 |
|  |  | hsa-miR-106a* | 3 |
| SOX11 | YA > MA | hsa-miR-9 | 3 |
|  |  | hsa-miR-518a-3p | 5 |
|  |  | hsa-miR-33b | 5 |
|  |  | hsa-miR-33b* | 3 |
|  |  | hsa-miR-210 | 5 |
|  |  | hsa-miR-106a | 3 |
|  |  | hsa-miR-106b | 3 |
| SOX15 | YA < MA | hsa-miR-210 | 3 |
| SPAG11B | YA < MA | hsa-miR-9 | 3 |
| SPATA12 | YA < MA | hsa-miR-210 | 4 |
|  |  | hsa-miR-106a | 3 |
| SPRYD4 | YA < MA | hsa-miR-9 | 5 |
| SPTLC2 | YA > MA | hsa-miR-9 | 7 |
|  |  | hsa-miR-372 | 6 |
|  |  | hsa-miR-106a | 7 |
|  |  | hsa-miR-106b | 6 |
|  |  | hsa-miR-106b* | 3 |
|  |  | hsa-miR-18b | 5 |
|  |  | hsa-miR-18b* | 3 |
| SSR3 | YA > MA | hsa-miR-9 | 7 |
|  |  | hsa-miR-106a | 3 |
|  |  | hsa-miR-106b | 6 |
| ST13 | YA > MA | hsa-miR-106a | 5 |
|  |  | hsa-miR-106a* | 3 |
|  |  | hsa-miR-106b | 3 |
|  |  | hsa-miR-18b | 6 |
| ST8SIA4 | YA < MA | hsa-miR-9 | 5 |
|  |  | hsa-miR-372 | 3 |
|  |  | hsa-miR-106a | 3 |
|  |  | hsa-miR-106b | 5 |
|  |  | hsa-miR-18b | 5 |
| STARD13 | YA < MA | hsa-miR-9 | 5 |
| STX16 | YA < MA | hsa-miR-372 | 4 |
| STXBP4 | YA > MA | hsa-miR-33b | 3 |
|  |  | hsa-miR-106a | 3 |
|  |  | hsa-miR-106b | 5 |
| STYK1 | YA < MA | hsa-miR-18b | 5 |
| SUPT7L | YA < MA | hsa-miR-9 | 5 |
|  |  | hsa-miR-210 | 5 |
|  |  | hsa-miR-106a | 4 |
|  |  | hsa-miR-18b | 5 |
| SURF4 | YA < MA | hsa-miR-9 | 5 |
| SVOP | YA < MA | hsa-miR-9 | 3 |
| TARBP1 | YA < MA | hsa-miR-106a | 4 |
| TBC1D24 | YA < MA | hsa-miR-210 | 3 |
| TEX12 | YA < MA | hsa-miR-106a | 4 |
| TFPI2 | YA > MA | hsa-miR-33b | 5 |
|  |  | hsa-miR-106a | 4 |
|  |  | hsa-miR-106b | 3 |
| TGOLN2 | YA < MA | hsa-miR-9 | 5 |
|  |  | hsa-miR-518a-3p | 5 |
|  |  | hsa-miR-372 | 5 |
|  |  | hsa-miR-33b | 4 |
|  |  | hsa-miR-106a | 5 |
|  |  | hsa-miR-106a* | 3 |
|  |  | hsa-miR-106b | 6 |
|  |  | hsa-miR-18b | 3 |
| THAP6 | YA < MA | hsa-miR-9 | 7 |
|  |  | hsa-miR-372 | 6 |
|  |  | hsa-miR-106a | 7 |
|  |  | hsa-miR-106a* | 3 |
|  |  | hsa-miR-106b | 7 |
| TJP2 | YA > MA | hsa-miR-33b | 4 |
| TLN1 | YA > MA | hsa-miR-9 | 4 |
| TMEM104 | YA < MA | hsa-miR-518a-3p | 4 |
| TMEM109 | YA < MA | hsa-miR-9 | 5 |
|  |  | hsa-miR-372 | 3 |
|  |  | hsa-miR-106a | 4 |
|  |  | hsa-miR-18b | 3 |
| TMEM128 | YA < MA | hsa-miR-106a | 5 |
|  |  | hsa-miR-106b | 5 |
| TMEM129 | YA < MA | hsa-miR-9 | 4 |
| TMEM132B | YA < MA | hsa-miR-9 | 5 |
|  |  | hsa-miR-372 | 4 |
|  |  | hsa-miR-33b | 5 |
|  |  | hsa-miR-106a | 4 |
|  |  | hsa-miR-106b | 5 |
|  |  | hsa-miR-18b | 5 |
| TMEM140 | YA < MA | hsa-miR-9 | 5 |
|  |  | hsa-miR-372 | 5 |
| TMEM176A | YA < MA | hsa-miR-372 | 5 |
| TMEM18 | YA > MA | hsa-miR-106a | 3 |
|  |  | hsa-miR-106b | 5 |
| TMEM25 | YA < MA | hsa-miR-372 | 5 |
|  |  | hsa-miR-106a | 7 |
|  |  | hsa-miR-106b | 6 |
|  |  | hsa-miR-18b | 5 |
| TMEM41B | YA < MA | hsa-miR-33b | 5 |
| TMEM64 | YA > MA | hsa-miR-372 | 6 |
|  |  | hsa-miR-33b | 4 |
|  |  | hsa-miR-106a | 5 |
|  |  | hsa-miR-106a* | 3 |
|  |  | hsa-miR-106b | 5 |
|  |  | hsa-miR-18b | 3 |
| TMEM9B | YA < MA | hsa-miR-33b | 5 |
|  |  | hsa-miR-106a | 3 |
|  |  | hsa-miR-106b | 5 |
| TNFRSF10A | YA > MA | hsa-miR-372 | 6 |
|  |  | hsa-miR-106a | 6 |
|  |  | hsa-miR-106b | 6 |
| TNFSF11 | YA > MA | hsa-miR-372 | 4 |
|  |  | hsa-miR-106a | 4 |
|  |  | hsa-miR-106b | 6 |
|  |  | hsa-miR-106b* | 3 |
| TNFSF12 | YA < MA | hsa-miR-9 | 3 |
| TNS1 | YA < MA | hsa-miR-9 | 6 |
|  |  | hsa-miR-372 | 4 |
|  |  | hsa-miR-33b | 3 |
|  |  | hsa-miR-210 | 4 |
|  |  | hsa-miR-106a | 5 |
|  |  | hsa-miR-106b | 4 |
|  |  | hsa-miR-18b | 3 |
| TOX4 | YA < MA | hsa-miR-9 | 5 |
| TP53INP2 | YA > MA | hsa-miR-372 | 6 |
|  |  | hsa-miR-106a | 5 |
|  |  | hsa-miR-106b | 6 |
|  |  | hsa-miR-18b | 5 |
|  |  | hsa-miR-18b* | 3 |
| TPD52L3 | YA < MA | hsa-miR-372 | 5 |
| TPM1 | YA > MA | hsa-miR-33b | 3 |
| TPM3 | YA > MA | hsa-miR-9 | 4 |
|  |  | hsa-miR-372 | 4 |
|  |  | hsa-miR-33b | 4 |
|  |  | hsa-miR-33b* | 3 |
|  |  | hsa-miR-106a | 4 |
|  |  | hsa-miR-106b | 4 |
| TRAF4 | YA > MA | hsa-miR-372 | 5 |
|  |  | hsa-miR-106a | 5 |
|  |  | hsa-miR-106b | 5 |
| TRIM22 | YA < MA | hsa-miR-372 | 4 |
|  |  | hsa-miR-106a | 5 |
|  |  | hsa-miR-106b | 5 |
| TRIP11 | YA < MA | hsa-miR-372 | 7 |
|  |  | hsa-miR-106a | 6 |
|  |  | hsa-miR-106b | 8 |
|  |  | hsa-miR-106b* | 3 |
| TRUB1 | YA < MA | hsa-miR-9 | 5 |
|  |  | hsa-miR-372 | 4 |
|  |  | hsa-miR-106a | 4 |
|  |  | hsa-miR-106b | 5 |
|  |  | hsa-miR-18b | 5 |
| TSG101 | YA < MA | hsa-miR-372 | 5 |
|  |  | hsa-miR-33b | 4 |
|  |  | hsa-miR-106a | 6 |
|  |  | hsa-miR-106a* | 3 |
|  |  | hsa-miR-106b | 8 |
|  |  | hsa-miR-106b* | 3 |
| TTC27 | YA < MA | hsa-miR-33b | 3 |
| TTN | YA < MA | hsa-miR-9 | 3 |
|  |  | hsa-miR-106b | 4 |
| UBOX5 | YA < MA | hsa-miR-372 | 6 |
|  |  | hsa-miR-106a | 6 |
|  |  | hsa-miR-106a* | 3 |
|  |  | hsa-miR-106b | 5 |
| UBQLN1 | YA < MA | hsa-miR-9 | 6 |
|  |  | hsa-miR-106a | 3 |
|  |  | hsa-miR-106b | 3 |
|  |  | hsa-miR-18b | 3 |
| UGT3A1 | YA < MA | hsa-miR-106a | 4 |
| UPK1B | YA < MA | hsa-miR-372 | 6 |
|  |  | hsa-miR-106a | 6 |
|  |  | hsa-miR-106b | 6 |
| USF1 | YA < MA | hsa-miR-33b | 5 |
|  |  | hsa-miR-210 | 3 |
| USP10 | YA > MA | hsa-miR-106a | 3 |
| USP33 | YA < MA | hsa-miR-372 | 4 |
|  |  | hsa-miR-106a | 5 |
|  |  | hsa-miR-106b | 5 |
| USP45 | YA < MA | hsa-miR-9 | 3 |
|  |  | hsa-miR-372 | 4 |
|  |  | hsa-miR-33b | 4 |
|  |  | hsa-miR-106a | 5 |
|  |  | hsa-miR-106b | 3 |
|  |  | hsa-miR-18b | 3 |
| USP49 | YA < MA | hsa-miR-518a-3p | 3 |
|  |  | hsa-miR-372 | 4 |
|  |  | hsa-miR-106a | 3 |
|  |  | hsa-miR-106b | 3 |
| USPL1 | YA < MA | hsa-miR-33b | 3 |
| UTY | YA < MA | hsa-miR-106a | 4 |
| VAV2 | YA < MA | hsa-miR-372 | 4 |
|  |  | hsa-miR-106a | 4 |
|  |  | hsa-miR-106b | 4 |
| VENTX | YA < MA | hsa-miR-372 | 5 |
| VEZT | YA < MA | hsa-miR-9 | 4 |
|  |  | hsa-miR-33b | 3 |
|  |  | hsa-miR-106a | 4 |
|  |  | hsa-miR-106b | 5 |
|  |  | hsa-miR-106b* | 3 |
| VPS13A | YA < MA | hsa-miR-33b | 5 |
|  |  | hsa-miR-18b | 6 |
|  |  | hsa-miR-18b* | 3 |
| VPS45 | YA > MA | hsa-miR-106a | 4 |
| VPS54 | YA < MA | hsa-miR-18b | 7 |
| WDR21A | YA < MA | hsa-miR-18b | 5 |
| WDR33 | YA > MA | hsa-miR-9 | 3 |
|  |  | hsa-miR-372 | 3 |
|  |  | hsa-miR-33b | 3 |
|  |  | hsa-miR-106a | 4 |
|  |  | hsa-miR-106a* | 3 |
|  |  | hsa-miR-106b | 4 |
|  |  | hsa-miR-18b | 3 |
| WDR91 | YA < MA | hsa-miR-9 | 3 |
|  |  | hsa-miR-106a | 3 |
|  |  | hsa-miR-106b | 3 |
| WHSC1 | YA < MA | hsa-miR-9 | 5 |
|  |  | hsa-miR-518a-3p | 5 |
|  |  | hsa-miR-372 | 4 |
|  |  | hsa-miR-33b | 3 |
|  |  | hsa-miR-106a | 5 |
|  |  | hsa-miR-106b | 5 |
|  |  | hsa-miR-18b | 5 |
| WIF1 | YA > MA | hsa-miR-33b | 6 |
| WNK3 | YA < MA | hsa-miR-372 | 5 |
|  |  | hsa-miR-106a | 6 |
|  |  | hsa-miR-106a* | 3 |
|  |  | hsa-miR-106b | 7 |
| XPC | YA > MA | hsa-miR-9 | 4 |
|  |  | hsa-miR-106a | 3 |
|  |  | hsa-miR-106b | 3 |
| XRN2 | YA > MA | hsa-miR-9 | 5 |
| YTHDC1 | YA > MA | hsa-miR-372 | 5 |
|  |  | hsa-miR-33b | 3 |
|  |  | hsa-miR-106a | 6 |
|  |  | hsa-miR-106a* | 3 |
|  |  | hsa-miR-106b | 6 |
| YWHAZ | YA > MA | hsa-miR-372 | 5 |
|  |  | hsa-miR-106a | 4 |
|  |  | hsa-miR-106b | 4 |
| ZBTB39 | YA < MA | hsa-miR-9 | 6 |
|  |  | hsa-miR-106a | 3 |
|  |  | hsa-miR-106b | 5 |
| ZBTB4 | YA < MA | hsa-miR-9 | 5 |
|  |  | hsa-miR-372 | 7 |
|  |  | hsa-miR-106a | 7 |
|  |  | hsa-miR-106b | 7 |
|  |  | hsa-miR-18b | 6 |
| ZC3H11A | YA < MA | hsa-miR-372 | 6 |
|  |  | hsa-miR-106b | 3 |
| ZC3H14 | YA > MA | hsa-miR-9 | 5 |
|  |  | hsa-miR-372 | 5 |
|  |  | hsa-miR-33b | 3 |
|  |  | hsa-miR-106a | 5 |
|  |  | hsa-miR-106b | 5 |
| ZC3H6 | YA < MA | hsa-miR-9 | 3 |
|  |  | hsa-miR-372 | 6 |
|  |  | hsa-miR-33b | 5 |
|  |  | hsa-miR-106a | 6 |
|  |  | hsa-miR-106b | 3 |
|  |  | hsa-miR-18b | 6 |
| ZC3HAV1 | YA < MA | hsa-miR-9 | 5 |
|  |  | hsa-miR-33b | 5 |
|  |  | hsa-miR-18b | 4 |
| ZDHHC21 | YA < MA | hsa-miR-106a | 4 |
| ZDHHC5 | YA < MA | hsa-miR-9 | 5 |
| ZFP36L1 | YA < MA | hsa-miR-518a-3p | 3 |
|  |  | hsa-miR-33b | 3 |
|  |  | hsa-miR-106a | 3 |
|  |  | hsa-miR-18b | 4 |
| ZFYVE9 | YA > MA | hsa-miR-372 | 5 |
|  |  | hsa-miR-106a | 6 |
|  |  | hsa-miR-106b | 8 |
|  |  | hsa-miR-106b* | 3 |
| ZMAT5 | YA > MA | hsa-miR-106a | 3 |
| ZMPSTE24 | YA < MA | hsa-miR-33b | 3 |
|  |  | hsa-miR-106a | 3 |
|  |  | hsa-miR-106b | 3 |
| ZNF10 | YA < MA | hsa-miR-9 | 5 |
|  |  | hsa-miR-33b | 3 |
|  |  | hsa-miR-106a | 4 |
|  |  | hsa-miR-18b | 5 |
| ZNF167 | YA < MA | hsa-miR-106a | 3 |
| ZNF248 | YA < MA | hsa-miR-9 | 5 |
| ZNF25 | YA < MA | hsa-miR-372 | 7 |
|  |  | hsa-miR-106a | 6 |
|  |  | hsa-miR-106b | 7 |
|  |  | hsa-miR-18b | 5 |
| ZNF282 | YA < MA | hsa-miR-9 | 3 |
|  |  | hsa-miR-518a-3p | 5 |
| ZNF343 | YA < MA | hsa-miR-33b | 5 |
|  |  | hsa-miR-18b | 5 |
| ZNF468 | YA > MA | hsa-miR-372 | 5 |
|  |  | hsa-miR-106a | 5 |
|  |  | hsa-miR-106b | 5 |
| ZNF485 | YA < MA | hsa-miR-33b | 4 |
| ZNF493 | YA < MA | hsa-miR-18b | 5 |
| ZNF544 | YA > MA | hsa-miR-33b | 3 |
| ZNF594 | YA < MA | hsa-miR-372 | 5 |
|  |  | hsa-miR-106a | 5 |
|  |  | hsa-miR-106b | 5 |
| ZNF670 | YA > MA | hsa-miR-518a-3p | 4 |
|  |  | hsa-miR-106a | 3 |
|  |  | hsa-miR-106b | 5 |
| ZNF680 | YA < MA | hsa-miR-33b | 3 |
|  |  | hsa-miR-106a | 4 |
|  |  | hsa-miR-106b | 6 |
| ZNF721 | YA < MA | hsa-miR-106b | 3 |
| ZZEF1 | YA < MA | hsa-miR-518a-3p | 5 |
|  |  | hsa-miR-372 | 4 |
|  |  | hsa-miR-106a | 4 |
|  |  | hsa-miR-106b | 4 |
