# Supplementary material for: A Set of miRNAs, Their Gene and Protein Targets and Stromal Genes Distinguish Early from Late Onset ER Positive Breast Cancer
Source: PLoS One. 2016 May 6;11(5):e0154325. doi: 10.1371/journal.pone.0154325 (PMC4859528; doi:10.1371/journal.pone.0154325)
Supplement: S2 Table — (DOC) [file pone.0154325.s002.doc]

**S2 Table.** Association between mRNAs and tumors clinicopathological characteristics.

| **mRNA** | | **Tumor size** | | | | | | | | **TNM** | | | | | | | | | **Lymph node** | | | | |
| --- | --- | --- | --- | --- | --- | --- | --- | --- | --- | --- | --- | --- | --- | --- | --- | --- | --- | --- | --- | --- | --- | --- | --- |
|  |  | **< 2 cm** | | | | **≥ 2 cm** | | | **P** | **I e II** | | | **III e IV** | | | **P** | | | **negative** | **positive** | | **P** | |
| ***BCL7*** | |  | | | |  | | |  |  | | |  | | |  | | |  |  | |  | |
| Under | | 10 (67%) | | | | 5 (33%) | | | 0.46 | 11 (73%) | | | 4 (27%) | | | 1 | | | 7 (64%) | 4 (36%) | | 1 | |
| Over | | 7 (50%) | | | | 7 (50%) | | |  | 11 (79%) | | | 3 (21%) | | |  |  |  | 9 (69%) | 4 (31%) | |  |  |
| ***ESR1*** | |  | | | |  | | |  |  | | |  | | |  | | |  |  | |  | |
| Under | | 7 (58%) | | | | 5 (42%) | | | 1 | 7 (58%) | | | 5 (42%) | | | 0.09 | | | 6 (60%) | 4 (40%) | | 0.67 | |
| Over | | 10 (59%) | | | | 7 (41%) | | |  | 15 (88%) | | | 2 (12%) | | |  |  |  | 10 (71%) | 4 (29%) | |  |  |
| ***YWHAZ*** | |  | | | |  | | |  |  | | |  | | |  | | |  |  | |  | |
| Under | | 8 (53%) | | | | 7 (47%) | | | 0.71 | 10 (67%) | | | 5 (33%) | | | 0.39 | | | 10 (77%) | 3 (23%) | | 0.39 | |
| Over | | 9 (64%) | | | | 5 (36%) | | |  | 12 (86%) | | | 2 (14%) | | |  |  |  | 6 (54%) | 5 (46%) | |  |  |
| ***DUSP8*** | |  | | | |  | | |  |  | | |  | | |  | | |  |  | |  | |
| Under | | 7 (58%) | | | | 5 (42%) | | | 1 | 7 (58%) | | | 5 (42%) | | | 0.09 | | | 5 (62%) | 3 (38%) | | 1 | |
| Over | | 10 (59%) | | | | 7 (41%) | | |  | 15 (88%) | | | 2 (12%) | | |  |  |  | 11 (69%) | 5 (31%) | |  |  |
| ***PIGS*** | |  | | | |  | | |  |  | | |  | | |  | | |  |  | |  | |
| Under | | 7 (50%) | | | | 7 (50%) | | | 0.46 | 10 (71%) | | | 4 (27%) | | | 0.68 | | | 8 (67%) | 4 (33%) | | 1 | |
| Over | | 10 (67%) | | | | 5 (33%) | | |  | 12 (80%) | | | 3 (20%) | | |  |  |  | 8 (67%) | 4 (33) | |  |  |
| ***PARP12*** | |  | | | |  | | |  |  | | |  | | |  | | |  |  | |  | |
| Under | | 9 (69%) | | | | 4 (31%) | | | 0.45 | 9 (69%) | | | 4 (31%) | | | 0.67 | | | 4 (40%) | 6 (60%) | | **0.03*** | |
| Over | | 8 (50%) | | | | 8 (50%) | | |  | 13 (81%) | | | 3 (19%) | | |  |  |  | 12 (86%) | 2 (14%) | |  |  |
| ***DUSP2*** | |  | | | |  | | |  |  | | |  | | |  | | |  |  | |  | |
| Under | | 12 (63%) | | | | 7 (37%) | | | 0.69 | 16 (84%) | | | 3 (16%) | | | 0.19 | | | 10 (67%) | 5 (33%) | | 1 | |
| Over | | 5 (50%) | | | | 5 (50%) | | |  | 6 (60%) | | | 4 (40%) | | |  |  |  | 6 (67%) | 3 (33%) | |  |  |
| ***RRS6KA1*** | | | |  |  | |  | | | |  | | |  | | |  |  | | |  | |  |
| Under | | 7 (54%) | | | | 6 (46%) | | | 0.72 | 8 (61%) | | | 5 (39%) | | | 0.19 | | | 7 (78%) | 2 (22%) | | 0.66 | |
| Over | | 10 (62%) | | | | 6 (38%) | | |  | 14 (87%) | | | 2 (13%) | | |  |  |  | 9 (60%) | 6 (40%) | |  |  |
|  |  | |  | | | | |  | | | |  | | |  | | | | | | | | |

#under expression of mRNAs (under); over expression of mRNAs (over) in the YA-BC as compared to MA-BC group
