# Supplementary material for: A Set of miRNAs, Their Gene and Protein Targets and Stromal Genes Distinguish Early from Late Onset ER Positive Breast Cancer
Source: PLoS One. 2016 May 6;11(5):e0154325. doi: 10.1371/journal.pone.0154325 (PMC4859528; doi:10.1371/journal.pone.0154325)
Supplement: S3 Table — (DOC) [file pone.0154325.s003.doc]

**S3 Table.** Biological processes associated with 21 interconnected protein.

| Biological process | Protein number | Protein Name | p-value_fdr |
| --- | --- | --- | --- |
| positive regulation of cellular process | 17 | NDRG1, STAT5A, PXN, RAF1, ESR1, YWHAZ, YWHAB, PRSKA1, PRSKA2, PRSKA3, EIF4E, EIFAEBP1, STMN1, NRG1, PARP1, RAD50, CDKN1B | 2.54E-6 |
| enzyme linked receptor protein signaling pathway | 16 | NDRG1, STAT5A, PXN, RAF1, ESR1, YWHAZ, YWHAB, PRSKA1, PRSKA2, PRSKA3, EIF4E, EIFAEBP1, STMN1, NRG1, PARP1, RAD50, CDKN1B | 1.05E-13 |
| multicellular organismal development | 16 | NDRG1, STAT5A, PXN, RAF1, ESR1, YWHAZ, YWHAB, PRSKA1, PRSKA2, PRSKA3, EIF4E, EIFAEBP1, NRG1, PARP1, RAD50, CDKN1B | 2.42E-5 |
| developmental process | 16 | STAT5A, PXN, PRSKB1, PRSKA1, PRSKA3, BCL2L1, RAF1,SRC, YWHAB, ESR1, CDKN1B, NDRG1, GYS1, PARP1, EIF4E, EIFAEBP1 | 9.78E-5 |
| cell cycle | 13 | YWHAZ, YWHAB, PRSKB1, PRSKA1, PRSKA3, EIF4E, EIFAEBP1, BCL2L1, SRC, RAD50, MDM2, CTNNB1,STMN1 | 5.84E-8 |
| regulation of apoptotic process | 13 | LCK, STAT5A, BCL2L1, YWHAB, YWHAZ, MDM2, RAF1, ESR1, PRSKA1, PRSKA2, PRSKA3, NRG1, CDKN1B | 8.34E-8 |
| regulation of metabolic process | 12 | RAF1, BCL2L1, PXN, EIF4E, EIFAEBP1, RAD50, PARP1, YWHAB, PRSKA1, PRSKA3, NRG1, PRSKB1 | 2.4E-5 |
| response to stress | 12 | PXN, SRC, PRSKB1, PRSKA1, PRSKA2, PRSKA3, YWHAB, EIFAEBP1, CTNNB1, NDRG1, NRG1, PARP1 | 1.52E-3 |
| neurotrophin signaling pathway | 10 | RAF1, YWHAB, PRSKA1, PRSKA2, NRG1, CDKN1B, MDM2,SRC, LCK | 4.37E-10 |
| ERBB signaling pathway | 8 | LCK, PXN, RAF1, YWHAB, CDKN1B, SRC, NRG1, MDM2 | 3.56E-8 |
| cell proliferation | 7 | RAF1, BCL2L1, SRC, ESR1, PRSKA1, CTNNB1, NRG1 | 2.77E-3 |
