# Supplementary material for: A Set of miRNAs, Their Gene and Protein Targets and Stromal Genes Distinguish Early from Late Onset ER Positive Breast Cancer
Source: PLoS One. 2016 May 6;11(5):e0154325. doi: 10.1371/journal.pone.0154325 (PMC4859528; doi:10.1371/journal.pone.0154325)
Supplement: S4 Table — (DOC) [file pone.0154325.s004.doc]

**S4 Table.** Association between proteins and tumors clinicopathological characteristics.

| **Proteins** | | | **Histological grade** | | | | | | | **Tumor size** | | | | | | | | | | | **TNM** | | | | | | **KI67** | | | | | | | | **Lymph node** | | | | |  |
| --- | --- | --- | --- | --- | --- | --- | --- | --- | --- | --- | --- | --- | --- | --- | --- | --- | --- | --- | --- | --- | --- | --- | --- | --- | --- | --- | --- | --- | --- | --- | --- | --- | --- | --- | --- | --- | --- | --- | --- | --- |
|  |  |  | **1 e 2** | **3** | | | | **P** | | **< 2 cm** | | | | **≥ 2 cm** | | **P** | | **I e II** | | | **III e IV** | | | | **P** | | **negative** | | | | **positive** | | **P** | | **negative** | | | **positive** | **P** |  |
| **RAF** | | |  |  | | | |  | |  | | | |  | |  | |  | | |  | | | |  | |  | | | |  | |  | |  | | |  |  |  |
| Under | | | 19 (76%) | 6 (24%) | | | | 0.065 | | 20 (77%) | | | | 6 (23%) | | 0.07 | | 25 (89%) | | | 3 (11%) | | | | **0.02*** | | 10 (38%) | | | | 16 (62%) | | 0.33 | | 18 (78%) | | | 5 (22%) | 1 |  |
| Over | | | 9 (47%) | 9 (41%) | | | |  |  | 11 (50%) | | | | 11 (50%) | |  |  | 13 (59%) | | | 9 (41%) | | | |  |  | 4 (21%) | | | | 15 (79%) | |  |  | 13 (76%) | | | 4 (24%) |  |  |
| **BCL2L1** | | |  |  | | | |  | |  | | | |  | |  | |  | | |  | | | |  | |  | | | |  | |  | |  | | |  |  |  |
| Under | | | 7 (70%) | 3 (30%) | | | | 0.43 | | 8 (67%) | | | | 4 (33%) | | 0.47 | | 10 (83%) | | | 2 (17%) | | | | 0.25 | | 1 (10%) | | | | 9 (90%) | | 0.35 | | 8 (89%) | | | 1 (11%) | 0.61 |  |
| Over | | | 8 (50%) | 8 (50%) | | | |  |  | 9 (50%) | | | | 9 (50%) | |  |  | 11 (61%) | | | 7 (39%) | | | |  |  | 5 (31%) | | | | 11 (69%) | |  |  | 10 (71%) | | | 4 (29%) |  |  |
| **EIF4E** | | |  |  | | | |  | |  | | | |  | |  | |  | | |  | | | |  | |  | | | |  | |  | |  | | |  |  |  |
| Under | | | 22 (76%) | 7 (24%) | | | | **0.03*** | | 18 (62%) | | | | 11 (38%) | | 0.76 | | 23 (74%) | | | 8 (26%) | | | | 1 | | 9 (35%) | | | | 17 (65%) | | 0.74 | | 15 (71%) | | | 6 (29%) | 0.48 |  |
| Over | | | 6 (40%) | 9 (60%) | | | |  |  | 13 (68%) | | | | 6 (32%) | |  |  | 15 (79%) | | | 4 (21%) | | | |  |  | 5 (26%) | | | | 14 (74%) | |  |  | 16 (84%) | | | 3 (16%) |  |  |
| **STAT5** | | |  |  | | | |  | |  | | | |  | |  | |  | | |  | | | |  | |  | | | |  | |  | |  | | |  |  |  |
| Under | | | 6 (50%) | 6 (50%) | | | | 0.69 | | 5 (39%) | | | | 8 (61%) | | 0.14 | | 5 (38%) | | | 8 (62%) | | | | **0.002*** | | 4 (40%) | | | | 6 (60%) | | 0.16 | | 7 (78%) | | | 2 (22%) | 1 |  |
| Over | | | 15 (64%) | 5 (36%) | | | |  |  | 12 (71%) | | | | 5 (29%) | |  |  | 16 (94%) | | | 1 (6%) | | | |  |  | 2 (13%) | | | | 14 (88%) | |  |  | 11 (79%) | | | 3 (21%) |  |  |
| **PARP1** | | |  |  | | | |  | |  | | | |  | |  | |  | | |  | | | |  | |  | | | |  | |  | |  | | |  |  |  |
| Under | | | 4 (50%) | 4 (50%) | | | | 0.68 | | 8 (80%) | | | | 2 (20%) | | 0.12 | | 7 (70%) | | | 3 (30%) | | | | 1 | | 1 (11%) | | | | 8 (89%) | | 0.38 | | 4 (50%) | | | 4 (50%) | **0.03*** |  |
| Over | | | 11 (61%) | 7 (39%) | | | |  |  | 9 (45%) | | | | 11 (55%) | |  |  | 14 (70%) | | | 6 (30%) | | | |  |  | 5 (29%) | | | | 12 (71%) | |  |  | 14 (93%) | | | 1 (7%) |  |  |
| **ESR1** | | |  |  | | | |  | |  | | | |  | |  | |  | | |  | | | |  | |  | | | |  | |  | |  | | |  |  |  |
| Under | | | 9 (53%) | 8 (47%) | | | | 0.68 | | 10 (59%) | | | | 7 (41%) | | 1 | | 12 (71%) | | | 5 (29%) | | | | 1 | | 3 (19%) | | | | 13 (81%) | | 0.64 | | 9 (64%) | | | 5 (36%) | 0.12 |  |
| Over | | | 6 (67%) | 3 (33%) | | | |  |  | 7 (54%) | | | | 6 (46%) | |  |  | 9 (69%) | | | 4 (31%) | | | |  |  | 3 (30%) | | | | 7 (70%) | |  |  | 9 (100%) | | | 0 (0%) |  |  |
| **RPS6KA1** | | |  |  | | | |  | |  | | | |  | |  | |  | | |  | | | |  | |  | | | |  | |  | |  | | |  |  |  |
| Under | | | 4 (44%) | 5 (56%) | | | | 0.42 | | 2 (22%) | | | | 7 (78%) | | **0.02*** | | 6 (67%) | | | 3 (33%) | | | | 1 | | 3 (38%) | | | | 5 (63%) | | 0.33 | | 4 (50%) | | | 4 (50%) | **0.03*** |  |
| Over | | | 11 (65%) | 6 (35%) | | | |  |  | 15 (75%) | | | | 6 (29%) | |  |  | 15 (71%) | | | 6 (29%) | | | |  |  | 3 (17%) | | | | 15 (83%) | |  |  | 14 (93%) | | | 1 (7%) |  |  |
| **WHAZ** | |  | | | |  |  | | | |  | |  | |  | | | |  | | |  | |  | | | |  | |  | |  | | | |  |  | | | |
| Negativo | | | 7 (70%) | 3 (30%) | | | | 0.43 | | 15 (65%) | | | | 8 (35%) | | 1 | | 10 (77%) | | | 3 (23%) | | | | 0.69 | | 2 (20%) | | | | 8 (80%) | | 1 | | 9 (90%) | | | 1 (10%) | 0.34 |  |
| Positivo | | | 8 (50%) | 8 (50%) | | | |  |  | 16 (64%) | | | | 9 (36%) | |  |  | 11 (65%) | | | 6 (35%) | | | |  |  | 4 (25%) | | | | 12 (75%) | |  |  | 9 (69%) | | | 4 (31%) |  |  |
|  |  | | | |  | |  | |  | | |  | | |  | |  | | |  | | |  | | |  | | |  | | |  | |  | | | | | | |

#under expression of proteins (under); over expression of proteins (over) in the YA-BC as compared to MA-BC group.
