# Supplementary material for: A Set of miRNAs, Their Gene and Protein Targets and Stromal Genes Distinguish Early from Late Onset ER Positive Breast Cancer
Source: PLoS One. 2016 May 6;11(5):e0154325. doi: 10.1371/journal.pone.0154325 (PMC4859528; doi:10.1371/journal.pone.0154325)
Supplement: S5 Table — (DOC) [file pone.0154325.s005.doc]

**S5 Table.** Biological stromal genes functions of of YA-BC and MA-BC tumors microenvironments (*Webgestalt*).

| ***Gene Symbol*** | **Gene name** |
| --- | --- |
| **Metabolism (C=1130; O=14; E=7.02; R=1.99; rawP=0.0118; adjP=0.0924)** | |
| *IDI2* | *isopentenyl-diphosphate delta isomerase 2* |
| *OTC* | *ornithine carbamoyltransferase* |
| *POLR1D* | *polymerase (RNA) I polypeptide D 16kDa* |
| *FUT9* | *fucosyltransferase 9 (alpha (1,3) fucosyltransferase)* |
| *ALG13* | *asparagine-linked glycosylation 13 homolog (S. cerevisiae)* |
| *UQCRQ* | *ubiquinol-cytochrome c reductase, complex III subunit VII, 9.5kDa* |
| *PIGQ* | *phosphatidylinositol glycan anchor biosynthesis, class Q* |
| *RDH16* | *retinol dehydrogenase 16 (all-trans)* |
| *ALDH1A3* | *aldehyde dehydrogenase 1 family, member A3* |
| *NDUFS5* | *NADH dehydrogenase (ubiquinone) Fe-S protein 5 15kDa (NADH-coenzyme Q reductase)* |
| *APIP* | *APAF1 interacting protein* |
| *B4GALT4* | *UDP-Gal:betaGlcNAc beta 1,4- galactosyltransferase, polypeptide 4* |
| *PDHX* | *pyruvate dehydrogenase complex, component X* |
| *AKR1C4* | *aldo-keto reductase family 1, member C4 (chlordecone reductase; 3-alpha hydroxysteroid dehydrogenase, type I; dihydrodiol dehydrogenase 4)* |
| **Glycosphingolipid - (C=26; O=2; E=0.16; R=12.38; rawP=0.0113; adjP=0.0924)** | |
| *B4GALT4* | *UDP-Gal:betaGlcNAc beta 1,4- galactosyltransferase, polypeptide 4* |
| *FUT9* | *fucosyltransferase 9 (alpha (1,3) fucosyltransferase)* |
| **Focal adhesion  (C=200; O=5; E=1.24; R=4.02; rawP=0.0085; adjP=0.0924)** | |
| *MAPK8* | *mitogen-activated protein kinase 8* |
| *PAK3* | *p21 protein (Cdc42/Rac)-activated kinase 3* |
| *COL11A2* | *collagen, type XI, alpha 2* |
| *MYL5* | *myosin, light chain 5, regulatory* |
| *IGF1* | *insulin-like growth factor 1 (somatomedin C)* |
| **Metabolism of xenobiotic by cytochrome P450 (C=71; O=3; E=0.44; R=6.80; rawP=0.0099; adjP=0.0924)** | |
| *GSTK1* | *glutathione S-transferase kappa 1* |
| *ALDH1A3* | *aldehyde dehydrogenase 1 family, member A3* |
| *AKR1C4* | *aldo-keto reductase family 1, member C4 (chlordecone reductase; 3-alpha hydroxysteroid dehydrogenase, type I; dihydrodiol dehydrogenase 4)* |
| **Cancer (C=326; O=6; E=2.03; R=2.96; rawP=0.0168; adjP=0.0987)** | |
| *MAPK8* | *mitogen-activated protein kinase 8* |
| *HSP90B1* | *heat shock protein 90kDa beta (Grp94), member 1* |
| *EGLN1* | *egl nine homolog 1 (C. elegans)* |
| *SHH* | *sonic hedgehog* |
| *GLI1* | *GLI family zinc finger 1* |
| *IGF1* | *insulin-like growth factor 1 (somatomedin C)* |
| **Receptor citocin-citocin interaction (C=265; O=5; E=1.65; R=3.04; rawP=0.0255; adjP=0.1198)** | |
| *CCL3L3* | *chemokine (C-C motif) ligand 3-like 3* |
| *IFNA8* | *interferon, alpha 8* |
| *CCL27* | *chemokine (C-C motif) ligand 27* |
| *CCL18* | *chemokine (C-C motif) ligand 18 (pulmonary and activation-regulated)* |
| *AMH* | *anti-Mullerian hormone* |
